# Conservation agriculture buffers persistent carbon loss under warming by microbial adaptation

**DOI:** 10.1093/ismejo/wrag224

**Published:** 2026-08-30

**Authors:** Jun Ling, Wolfgang Wanek, Kyle Mason-Jones, Jennifer A J Dungait, Luiz A Domeignoz-Horta, Ruixing Hou, Yakov Kuzyakov, Fusuo Zhang, Jing Tian

**Affiliations:** Key Laboratory of Ecosystem Network Observation and Modeling, Institute of Geographic Sciences and Natural Resources Research, Chinese Academy of Sciences, 100101 Beijing, China; State Key Laboratory of Nutrient Use and Management, College of Resources and Environmental Sciences, China Agricultural University, 100193 Beijing, China; Department of Microbiology and Ecosystem Science, Division of Terrestrial Ecosystem Research, Center of Microbiology and Environmental Systems Science, University of Vienna, 1010 Vienna, Austria; Soil Microbial Interactions, Department of Geoscience, University of Tübingen, 72076 Tübingen, Germany; Cluster of Excellence (EXC 3121): TERRA – Terrestrial Geo-Biosphere Interactions in a Changing World, University of Tübingen, 72076 Tübingen, Germany; College of Life and Environmental Sciences, University of Exeter, EX4 4QJ Exeter, United Kingdom; Carbon Management Centre, Scotland’s Rural College (SRUC), EH9 3JG Edinburgh, United Kingdom; Swiss Federal Institute for Forest, Snow and Landscape Research WSL, 8903 Birmensdorf, Switzerland; Université Paris-Saclay, INRAE, AgroParisTech, UMR EcoSys, 91120 Palaiseau, France; Key Laboratory of Ecosystem Network Observation and Modeling, Institute of Geographic Sciences and Natural Resources Research, Chinese Academy of Sciences, 100101 Beijing, China; Departments of Soil Science of Temperate Ecosystems and Agricultural Soil Science, University of Göttingen, 37077 Göttingen, Germany; State Key Laboratory of Nutrient Use and Management, College of Resources and Environmental Sciences, China Agricultural University, 100193 Beijing, China; State Key Laboratory of Nutrient Use and Management, College of Resources and Environmental Sciences, China Agricultural University, 100193 Beijing, China; Lead contact

**Keywords:** stable-isotope labeling, substrate use efficiency, soil organic carbon, metabolic plasticity, agroecosystem management

## Abstract

Microbial carbon use efficiency (CUE) governs soil carbon persistence under warming, with its magnitude highly dependent on how microbial metabolism adapts to varying substrates. How agricultural management mediates this response in agroecosystems remains poorly understood. To address this, we coupled ^18^O-H_2_O tracing of community-level CUE with ^13^C-probing of substrate utilization efficiency (SUE) from substrates differing in apparent bioavailability after 11 years of experimental warming under conventional and conservation agriculture, using glucose as a readily available carbohydrate and vanillin as a less accessible lignin-derived aromatic substrate. Warming stimulated glucose SUE under both management regimes, whereas vanillin SUE declined by 20% exclusively under conservation agriculture. This shift in relative anabolic efficiency (RAE, SUE_glucose_/SUE_vanillin_) was associated with warming-induced changes in CUE, primarily through changes in vanillin rather than glucose SUE. Concurrently, conservation agriculture improved substrate quality under warming, reflected by a reduced litter lignocellulose index. In response, microbial communities exhibited distinct functional adaptations, shifting toward copiotrophic taxa with higher rRNA operon copy numbers and a 16% increase in the anabolic-to-catabolic gene ratio, indicating enhanced microbial growth potential and greater investment for labile-carbon processing. Structural equation modeling further showed that shifts in substrate quality were a primary driver of RAE, mediated by changes in microbial life-history strategies under conservation agriculture. Together, these results provide a genome-resolved mechanistic framework showing that conservation agriculture enhances soil carbon persistence under warming by reshaping microbial relative anabolic efficiency and metabolic strategies, highlighting metabolic adaptation as a key mechanism regulating soil carbon stability in warming agroecosystems.

## Graphical abstract

**Graphical Abstract:**
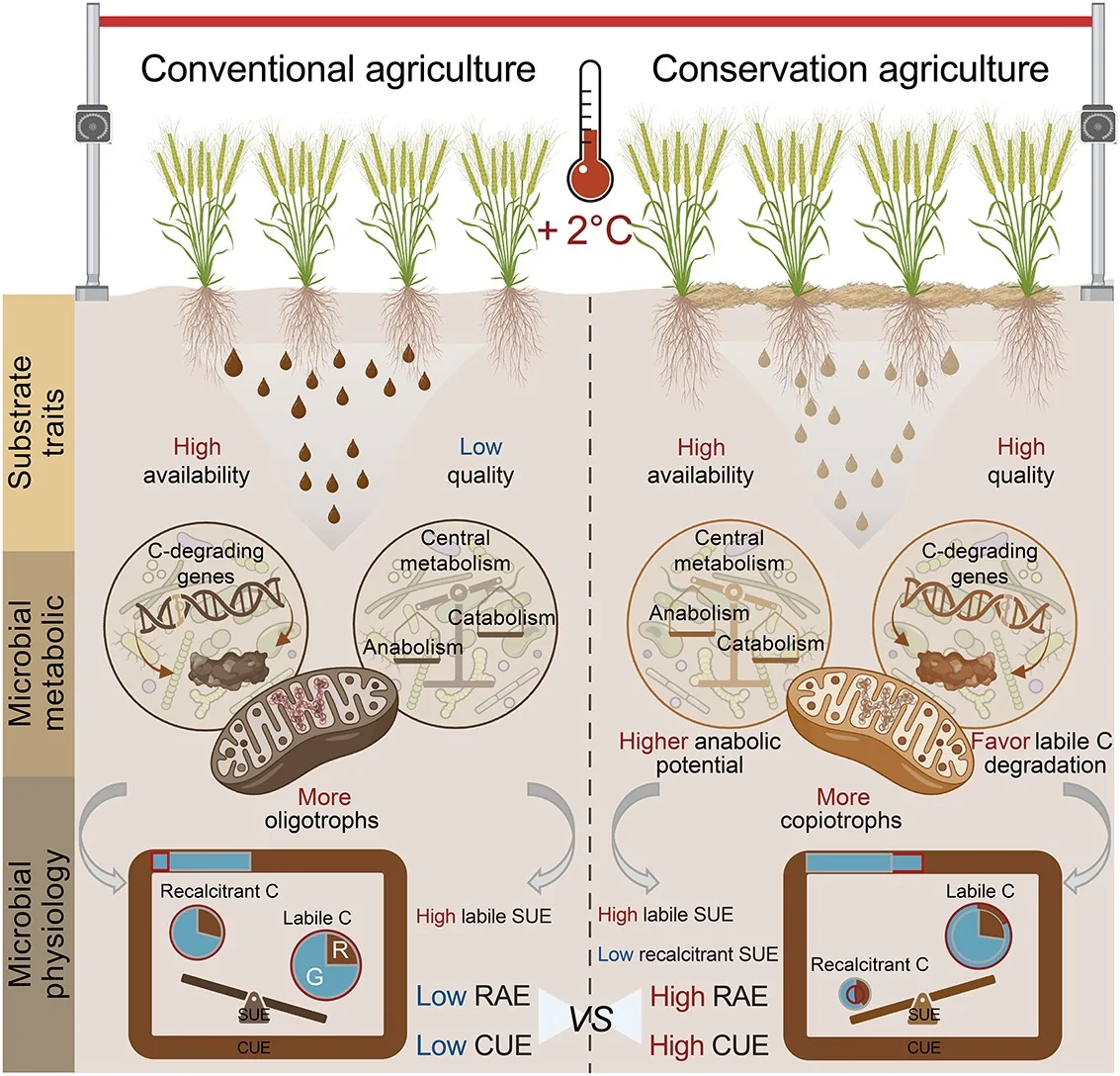

## Introduction

As primary agents of soil organic carbon (SOC) decomposition and major contributors to its formation, soil microorganisms play a seemingly paradoxical dual role in the global carbon (C) cycle [1, 2]. Microbial catabolic activities mineralize organic C and drive soil C loss to the atmosphere as CO_2_, whereas microbial anabolism converts assimilated C into microbial biomass–derived necromass that contributes to stable soil C pools [1]. Microbial carbon use efficiency (CUE) is an emergent physiological trait that integrates anabolic and catabolic processes and acts as a primary control on SOC formation [3]. Microorganisms with high CUE promote net C accrual and stabilization by converting a greater fraction of assimilated C into microbial biomass, eventually stimulating necromass formation [4]. Generally, warming stimulates microbial respiration more than growth, thereby reducing CUE, as elevated temperature increases maintenance costs and energy demands and thereby increases microbial respiration [5]. However, because of the dynamic nature and context dependence of substrate quality and availability [6], of microbial community composition and function [7, 8], and of physiological acclimation [9, 10], temperature-induced declines in CUE predicted solely from intrinsic physiological principles are not universal.

According to the substrate depletion hypothesis, long-term warming accelerates the consumption of labile-C pools in the absence of microbial acclimation [11] and selects for microbial communities that can utilize more stabilized C to fuel metabolic activities [5, 12]. This necessitates the expression of multiple additional catabolic genes for refractory C decomposition, which incurs larger energetic costs and thereby reduces microbial growth and CUE [13]. In the short term, two contrasting warming responses have been found: (i) following the rate–yield trade-off, warming increases organic matter decomposition and soil nutrient availability, favoring fast-growing copiotrophic microorganisms with higher ribosomal RNA gene copy numbers and lower microbial CUE [14, 15]. (ii) Under C-rich conditions, fast-growing microorganisms feeding on labile C sources invest less in exoenzyme secretion and supplementary metabolism, the reduced enzyme costs favoring C allocation to growth and increasing CUE [16]. Hence, substrate and resource modifications induced by warming may lead to inconsistent CUE responses across temporal scales. In addition to these substrate-driven processes, warming also triggers notable shifts in microbial abundance [11, 17], microbial community composition [18], functional and physiological traits [19], and growth rates [20], which potentially affect microbial C allocation and CUE. Soil microbiome responses to warmer temperatures are timescale dependent; short-term warming tends to intensify microbial activity, increasing enzymatic activities, decomposition, and soil respiration [21], whereas longer-term warming leads to substrate depletion and microbial thermal acclimation [11, 22], declines in fungal biomass [11, 23], and further shifts in physiological traits [24]. These dynamic interactions underscore the importance of considering resource-driven processes and microbial acclimation when evaluating the long-term effects of warming on soil C dynamics.

Soil management strategies designed to maximize microbial CUE could help to restore depleted farmland soil C stocks and regenerate soil health. Conservation agriculture is commonly proposed as a strategy to increase SOC sequestration, through reduced or zero tillage, crop residue retention, and crop rotations [25, 26]. Conservation agriculture is often assumed to enhance microbial CUE and thereby facilitate SOC sequestration, yet evidence from previous studies has been mixed [26, 27]. Several mechanisms that are not mutually exclusive could explain the lack of agreement. First, C-rich organic inputs from crop residues may increase population-level CUE through physiological changes, termed phenotype modification [28], while simultaneously selecting for populations with inherently lower CUE through trait filtering [27, 29], thereby reducing community-level efficiency. Second, conservation practices such as reduced tillage and cover cropping tend to concentrate hotspots of microbial activity in topsoil [30, 31], favoring the growth of copiotrophic microorganisms [32, 33], which can lead to increases in CUE due to relieved enzyme cost constraints and enhanced C allocation to growth [34] or to decreases in CUE through rate–yield trade-offs [15]. Third, macroaggregate formation and stabilization under reduced tillage increase macropore abundance and pore connectivity [35], improving soil microbial habitat quality where increased microbial resource acquisition benefits microbial assimilation processes and CUE [36]. With conservation agriculture now practiced on 12.5% of the world’s arable land [37], a better understanding of how climate change shapes soil microbial functions could advance forecasts of the climate change mitigation potential of alternative crop management [38].

Our overarching research question was how decadal warming interacts with agricultural management practices to alter the microbial utilization efficiency of substrates with contrasting bioavailability, through coupled changes in soil substrate–microbial trait space. Our specific hypotheses were as follows: (H1) decadal warming and agricultural management interact to shape soil microorganism–substrate dynamics, leading to changes in microbial substrate relative anabolic efficiency through thermal adaptation and substrate depletion. (H2) In contrast to conventional systems, conservation agriculture provides a sustained supply of high-quality substrates that offsets warming-induced resource depletion, thereby shifting the microbial adaptive trajectory. We hypothesize that this energetic subsidy alleviates the metabolic constraints of thermal stress, fostering a community dominated by copiotrophic strategies. This decadal adaptation reinforces a preference for labile carbon, allowing microorganisms to avoid recalcitrant C degradation and sustain higher growth efficiency under warming (H3). To test these hypotheses, we sampled topsoil from four experimental treatments (conservation vs. conventional management, with and without warming) established at the Yucheng experimental station, China, in 2009. We applied substrate-independent H_2_^18^O labeling to measure soil microbial CUE and substrate-dependent ^13^C-labeling with ^13^C-glucose and ^13^C-vanillin, respectively, to investigate soil microbial substrate use efficiency (SUE) for two contrasting substrates. Glucose was used as a readily available non-aromatic carbohydrate, whereas vanillin was used as a lignin-derived aromatic substrate with lower apparent bioavailability and greater metabolic processing requirements than glucose (Table S1). Edaphic conditions, substrate availability and quality, and microbial traits were quantified to elucidate the potential mechanisms driving the variation of microbial efficiencies.

## Materials and methods

### Site description and field experiment design

This long-term agricultural management experiment was initiated in 2003 at the Yucheng Comprehensive Experiment Station of the Chinese Academy of Sciences (36°50′N, 116°34′E). Subject to a temperate semi-humid monsoon climate, the site experiences an average annual temperature of 13.1°C and an annual rainfall of 561 mm. The soil is classified as a silt-loam Calcaric Fluvisol (World Reference Base for Soil Resources), with a particle size distribution of 12% sand, 22% clay, and 66% silt.

The cropping system consists of a double-cropping rotation of summer maize (*Zea mays* L.) and winter wheat (*Triticum aestivum* L.). This study contrasts a conservation agriculture system against conventional management, which involves post-harvest residue extraction coupled with mechanical soil inversion down to 10–15 cm to incorporate crop remnants. The conservation treatment is based on a no-till regime with a surface mulch of chopped maize straw (~5-cm fragments). For the wheat crop, all experimental plots received identical nutrient inputs (178 kg P ha^−1^ and 122 kg K ha^−1^ from compound fertilizers, together with a total of 285 kg N ha^−1^). In the conservation treatment, 47 kg N ha^−1^ of the total nitrogen input was supplied organically via mulched maize residues. All other management practices, including sowing and fertilization procedures, were identical across treatments.

A warming manipulation experiment was established in 2009 using 2 m × 2 m plots arranged in a randomized complete block design with four replicates per treatment. The experimental design included four treatment combinations derived from two cropping systems (conventional and conservation agriculture) and two temperature levels (ambient and +2°C warming). Active warming was achieved via overhead infrared heaters (Model MSR-2420; Kalglo Electronics Inc., Bethlehem, PA, USA), which were positioned at a height of roughly 3 m above the ground. On average, the infrared heaters increased soil temperature by ~2°C (Fig. S1), consistent with projected warming scenarios for the North China Plain under greenhouse gas emission trajectories reported by the Intergovernmental Panel on Climate Change [37]. Further details of the experimental design are described elsewhere [26].

**Figure 1:**
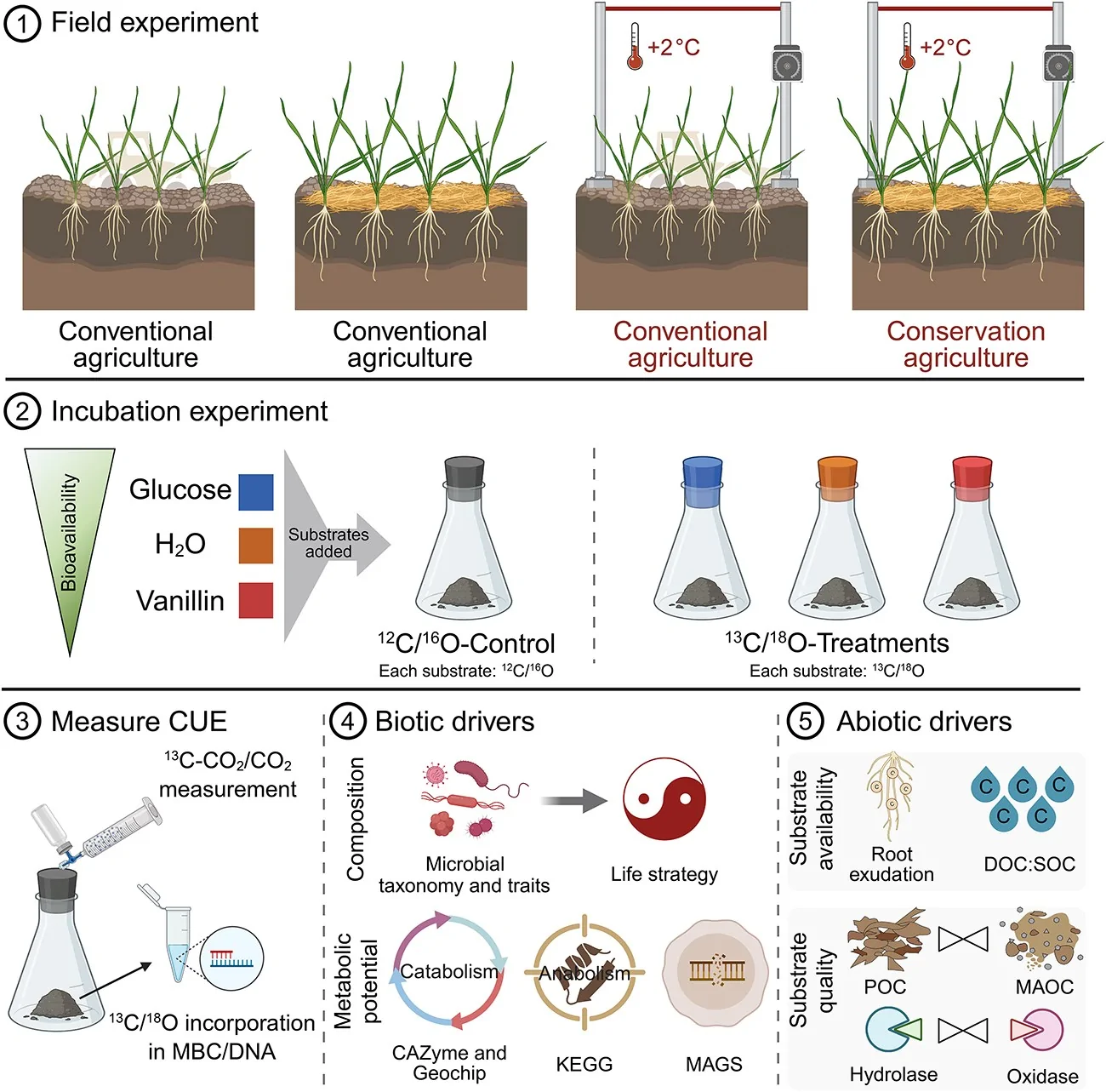
Overview of experimental design and key analyses. Samples were collected from a long-term management and warming field experiment, which had been established 11 years ago, in 2009. Soil cores were collected from four replicated plots. Microbial C use efficiency and the utilization efficiency of substrates varying in lability were evaluated using the ^18^O-H_2_O, ^13^C-glucose, and ^13^C-vanillin tracing approaches, respectively. The abundance of C-degrading gene families and pathways was quantified using metagenomics and quantitative microbial element cycling (QMEC) analysis. Substrate accessibility and quality were assessed using various C and enzyme-related metrics. CAZyme, carbohydrate-active enzyme; KEGG, Kyoto Encyclopedia of Genes and Genomes; MAGs, metagenome-assembled genomes; DOC, dissolved organic carbon; SOC, soil organic carbon; POC, particulate organic carbon; MAOC, mineral-associated organic carbon. Created in BioRender. Ling, J. (2026) https://BioRender.com/0yxwwi7.

In May 2021 (wheat maturation stage), five soil samples were collected from the surface layer (0–5 cm soil depth) with a soil corer in each of the 16 treatment plots after removal of any surface crop residues and mixed to form a composite sample. These composites were hand-sorted to remove roots and sieved (2-mm mesh). The homogenized field-moist soil was separated into three subsampling groups. One subsample was air-dried for analysis of SOC and soil C fractions. The second subsample was stored at 4°C for analysis of soil enzyme activities, dissolved organic carbon (DOC), and microbial C use efficiency. The third subsample was stored at −20°C for analysis of microbial taxonomic and functional community structure (Fig. 1).

### Soil microbial carbon substrate availability and quality

Soil C availability was evaluated using root exudation and the ratio of DOC to SOC [39]. Root-derived carbon inputs via exudation were obtained from a previously published dataset collected during the winter wheat growing season from October 2019 to May 2020 [26]. SOC contents were evaluated through the dry combustion technique (Vario EL III Elemental Analyzer; Elementar, Germany). DOC was extracted with 0.5 mol L^−1^ K_2_SO_4_ at 1:4 ratio of soil to extractant (w/v) by shaking (100 rev min^−1^) for 1 h on a shaker, and the extracts were filtered through 0.45-μm-pore-size membrane filters. The extracted organic C was measured using a TOC analyzer (Vario PYRO Cube, Hanau, Germany).

Given the range in structural complexity of potential microbial C substrates, we used two types of proxies to explore changes in substrate quality: the enzymatic lignocellulose index (LCI) and the ratio of mineral-associated organic carbon (MAOC) to particulate organic carbon (POC), expressed as MAOC:POC. Potential hydrolytic and oxidative enzyme activities were determined using a method described elsewhere [40], with detailed experimental procedures provided in Supplementary Note 1. In brief, β-1,4-glucosidase activity was measured using a 4-methylumbelliferone–based fluorometric assay, whereas polyphenol oxidase activity was measured using a l-3,4-dihydroxyphenylalanine–based spectrophotometric assay. The enzymatic LCI was calculated as the ratio of lnPPO to the sum of the lnPPO and lnBG [41]:


$${\mathrm{LCI}}_{\mathrm{enzyme}}=\frac{\mathrm{lnPPO}}{\mathrm{lnPPO}+\mathrm{lnBG}}$$

where lnPPO and lnBG indicate the natural-log-transformed activities of PPO and BG, respectively.

The hydrolase (BG) catalyzes the hydrolytic depolymerization of cellulose, whereas the oxidase (PPO) contributes to the breakdown of complex and recalcitrant substrates such as lignin. Therefore, the enzymatic LCI serves as an index of the relative availability of chemically complex SOC substrates: a higher LCI reflects a lower proportion of cellulose and a higher proportion of lignin, indicating lower substrate quality.

Soil samples were physically fractionated into MAOC (<53 μm) and POC (>53 μm) using a wet-sieving protocol described elsewhere [42]. Briefly, 20 g of sieved soil was chemically and mechanically dispersed using sodium hexametaphosphate solution (100 ml, 0.5%) together with glass beads under continuous shaking (18 h, ~200 rpm). The resulting slurry was rinsed with deionized water over a 53-μm sieve to separate the particulate (>53 μm) and mineral-associated (<53 μm) fractions. Carbon contents of the isolated fractions were subsequently determined using an elemental analyzer. The fractionation procedure achieved mean recovery rates of 98% for soil mass (range: 94%–102%) and 96% for carbon (range: 90%–112%).

### Soil microbial community structure and abundance

DNA was extracted using a PowerSoil DNA Kit (Qiagen) and subsequently sent to Majorbio (Shanghai, China) for bacterial 16S rRNA and fungal ITS community analyses. For bacteria, the V4–V5 hypervariable region of the 16S rRNA gene was amplified using the primer pair 515F (5′-GTGCCAGCMGCCGCGGTAA-3′) and 907R (5′-CCGTCAATTCMTTTRAGTTT-3′). For fungi, the internal transcribed spacer 2 (ITS2) region of the rRNA operon was amplified using primers gITS7F (5′-GTGARTCATCGARTCTTTG-3′) and ITS4R (5′-TCCTCCGCTTATTGATATGC-3′). Amplicons were sequenced using a NovaSeq System (Illumina) to generate 250-bp paired-end reads.

Data quality and analyses were performed using the DADA2 and QIIME 2 (v.2024.2) pipelines [43]. The DADA2 package was used to quality-filter sequences and infer amplicon sequence variants (ASVs). The resulting ASVs of bacteria were assigned taxonomically by mapping to the SILVA database (release 138.2) [44], and fungal taxonomy was assigned using the UNITE (v10.0) database [45]. To account for differences in sequencing depth among samples, all samples were rarefied at the minimum number of reads. To characterize how microbial community structure responded to warming and to management practices, we used the R packages *vegan* (v2.6) and *phyloseq* (v1.40.0). To calculate the ratio of oligotrophic to copiotrophic taxa among bacteria and fungi, the relative abundances of the most likely oligotrophic and copiotrophic taxa were summed separately. Details of taxa belonging to oligotrophic and copiotrophic taxa among bacteria and fungi are presented in Table S2. The 16S rRNA operon copy number was estimated based on the *rrn*DB database (version 5.9) [46]. For each sample, the community-level *rrn* copy number—a community-level aggregated trait—was calculated as the weighted mean of the estimated *rrn* copy number for each ASV, with weights based on their relative abundance, using Eq. (1) as follows:


(1) \begin{eqnarray*} \mathrm{community}-\mathrm{level}\ rrn\ \mathrm{copy}\ \mathrm{number}=\frac{\sum_{i=1}^N{S}_i}{\sum_{i=1}^N\frac{S_i}{n_i}} \end{eqnarray*}

where *N* is the number of ASVs in a sample, *S_i_* is the sequence abundance of ASV*_i_*, and *n_i_* is the estimated *rrn* copy number of ASV*_i_*.

Phospholipid fatty acid (PLFA) extractions were conducted to assess the biomass of soil bacteria and fungi, as well as its ratio (F:B) [47]. Briefly, a methanol–chloroform–citrate extraction system was used to recover lipids from 2 g of freeze-dried soil. Silicic acid chromatography facilitated the targeted retention of phospholipids, effectively discarding glycolipids and neutral lipids. Once isolated, the PLFAs were converted to fatty acid methyl esters (via saponification and methylation) for gas chromatographic analysis. To determine total bacterial biomass, we summed the PLFA concentrations attributed to actinobacteria, anaerobes, and both Gram-positive and Gram-negative taxa. Total fungal biomass was similarly derived by pooling the marker quantities for common fungi and AMF.

### Soil microbial life-history strategies and carbon cycling functions

DNA libraries were sequenced on a NovaSeq System (Illumina). Raw reads were quality-filtered, assembled, and annotated; metagenome-assembled genomes (MAGs) were reconstructed and refined as described in Supplementary Note 2. To complement taxonomy-based and rrnDB-derived life-history proxies, we estimated microbial genomic traits from metagenomic reads, assembled contigs, and recovered MAGs. Average genome size (AGS) and average 16S rRNA gene copy number (ACN) were estimated from unassembled quality-filtered metagenomic reads using the AGS–ACN approach [48]. For contig-derived traits, assembled contigs shorter than 500 bp were removed, protein-coding genes were predicted using Prokka, and nucleotide coding sequences were used for gRodon analysis. gRodon-derived traits included codon usage bias of highly expressed genes (CUBHE) and predicted minimal doubling time, with ribosomal protein genes used as highly expressed genes [49]. For MAG-derived traits, coding sequences from each MAG were analyzed using gRodon to estimate CUBHE and predicted minimal doubling time [50]. tRNA genes were predicted using tRNAscan-SE, excluding pseudo-tRNAs from the main counts [51]. MAG-level traits were then combined with MAG relative abundance to calculate community-weighted mean traits for each metagenomic sample. These read-, contig-, and MAG-derived metrics were interpreted as complementary genomic proxies for microbial life-history potential, rather than direct measurements of realized growth.

Predicted genes were functionally annotated against the CAZy and KEGG databases to characterize microbial substrate utilization and metabolic pathways. Gene abundances were normalized to transcripts per million. To complement metagenomic analyses and obtain absolute quantification of key C-cycling functions, functional genes were quantified using the Quantitative Microbial Elemental Cycling (QMEC) approach. Genes involved in the degradation of labile and recalcitrant C substrates were quantified, with gene copy numbers normalized to the 16S rRNA gene abundance. Given the higher accuracy of absolute quantification, QMEC data were used for functional gene analyses. The collective abundance of gene markers targeting cellulose hydrolysis, chitin hydrolysis, and lignin degradation was categorized as less-labile/structurally complex C degradation. In parallel, the labile-C degradation group was constituted by summing the functional genes associated with the hydrolysis of starch, hemicellulose, and pectin. Medium-quality MAGs were retained, taxonomically classified, and quantified by read mapping. Functional annotation of MAGs focused on carbohydrate-active enzymes and KEGG metabolic pathways to link microbial taxa with C utilization potential.

### Carbon use efficiency: ^18^O-water and ^13^C-substrate labeling method

CUE was quantified using stable isotope labeling with a short-term (48-h) incubation assay [52, 53]. Here, a dual stable-isotope labeling approach was applied: ^18^O-water was used to explore substrate-independent CUE, and ^13^C-glucose and ^13^C-vanillin were used to investigate substrate-specific CUE (SUE). Each of the field treatments contained four independent field plots, and the composite soil sample collected from each plot was treated as one biological replicate. Therefore, the CUE and SUE assays were conducted with four biological replicates per treatment (*n* = 4), corresponding to 16 independent soil samples in total. The short timescale of the assay was chosen to avoid microbial recycling of the labeled C prior to all procedures; soil samples were adjusted to 50% of their water-holding capacity and pre-incubated in the dark at 25°C for 1 week. For the ^18^O-water tracing method, two aliquots of 0.5 g (dry weight) of each soil sample were weighed into 2-ml falcon tubes. Following the conditions optimized in our pre-experiments, one of the aliquots was labeled to 40 at%^18^O in soil water by applying 39.5 μl ^18^O-water (97 at%) with a syringe. Parallel natural abundance controls were simultaneously prepared by incorporating the exact same volume of unlabeled H_2_O. The tubes were immediately transferred into 50-ml incubation vials and sealed with crimp caps. After 48-h incubation at 60% water-holding capacity, 12 ml of headspace gas was taken from each vial with a gas-tight syringe. The CO_2_ concentration was immediately determined using a gas chromatography system (GC-2010 Plus; SHIMADZU, Japan). Soil DNA was extracted from samples using a Qiagen DNeasy PowerSoil Kit (Venlo, Netherlands) following the manufacturer’s instructions. DNA samples were dehydrated at 60°C for 48 h in silver capsules to remove any moisture before isotope analysis. Subsequently, the ^18^O abundance and the total oxygen content in DNA were assayed by an Elementar PyroCube linked to an Isoprime VisION IRMS.

Microbial respiration (C_respiration_) was calculated using the amount of CO_2_-C produced during the 48-h incubation period using Eq. (2). Newly formed DNA was quantified using Eq. (3) based on the ^18^O excess, which was defined as the isotopic increase in the sample DNA over the natural background values of the control DNA. The flux of C to biomass production (C_growth_) was calculated by dividing the amount of MBC produced during the incubation by the incubation duration using Eq. (4).


(2) \begin{eqnarray*} {\mathrm{C}}_{\mathrm{respiration}}\left(\mathrm{\mu} \mathrm{g}\ \mathrm{C}\ {\mathrm{g}}^{-1}\ {\mathrm{h}}^{-1}\right)=\frac{D_{\mathrm{C}\mathrm{O}2}}{m_{\mathrm{soil}}\times t}\times \frac{p\times{V}_{\mathrm{h}\mathrm{s}}}{R\times T}\times M \end{eqnarray*}

(3) \begin{eqnarray*} {\mathrm{DNA}}_{\mathrm{produced}}\ \left(\mathrm{\mu} \mathrm{g}\right)={\mathrm{O}}_{\mathrm{total}}\times \frac{\mathrm{at}{\%}_{\mathrm{excess}}}{100}\times \frac{100}{\mathrm{at}{\%}_{\mathrm{label}}}\times \frac{100}{31.21} \end{eqnarray*}

(4) \begin{eqnarray*} {\mathrm{C}}_{\mathrm{g}\mathrm{rowth}}\ \left(\mathrm{\mu} \mathrm{g}\ \mathrm{C}\ {\mathrm{g}}^{-1}\ {\mathrm{h}}^{-1}\right)=\frac{f_{\mathrm{DNA}}\times{\mathrm{DNA}}_{\mathrm{produced}}}{m_{\mathrm{soil}}\times t} \end{eqnarray*}

In Eq. (2), *m*_soil_ is the dry weight of soil and *t* is the incubation time, *p* is the pressure (kPa) in the vial, *M* is the molecular mass of carbon (12.01 g mol^−1^), *R* is the universal gas constant (8.314 J mol^−1^), *T* is the temperature (295.15 K), *V*_hs_ is the volume (L) of the vial headspace, and *D*_CO2_ is the increase in CO_2_ concentration (ppm) during the 48-h incubation period. In Eq. (3), O_total_ (μg) is the total amount of O in the DNA sample, at%_excess_ is the difference in atomic percent ^18^O between the labeled and the non-labeled natural abundance control samples, and at%_label_ is the ^18^O enrichment of soil water. The average percent w/w of O in DNA is 31.21. *f*_DNA_ was obtained by dividing the MBC content of each sample by its corresponding DNA content.

The amount of C taken up by the microbial biomass (C_uptake_) was calculated as the sum of microbial respiration and growth.


(5) \begin{eqnarray*} {\mathrm{C}}_{\mathrm{uptake}}\ \left(\mathrm{\mu} \mathrm{g}\ \mathrm{C}\ {\mathrm{g}}^{-1}\ {\mathrm{h}}^{-1}\right)={\mathrm{C}}_{\mathrm{respiration}}+{\mathrm{C}}_{\mathrm{g}\mathrm{rowth}} \end{eqnarray*}

Subsequently, microbial ^18^O-derived CUE was calculated as follows:


(6) \begin{eqnarray*} \mathrm{CUE}=\frac{{\mathrm{C}}_{\mathrm{growth}}}{{\mathrm{C}}_{\mathrm{growth}}+{\mathrm{C}}_{\mathrm{respiration}}} \end{eqnarray*}

Microbial ^13^C-substrate use efficiency (SUE) was determined by tracing the uptake and mineralization of ^13^C-glucose (Glu) and ^13^C-vanillin (Van) [54–56]. We used glucose and vanillin as chemically contrasting plant litter–derived substrates that differ in apparent bioavailability: glucose represents a readily available non-aromatic carbohydrate released during cellulose decomposition, whereas vanillin represents a lignin-derived aromatic aldehyde with lower apparent bioavailability than glucose, as indicated by its moderate solubility, higher XLogP3/LogP, lower NOSC, and requirement for specialized aromatic–catabolic pathways before entry into central carbon metabolism (Table S1). Pre-incubated fresh soil aliquots (20 g dry weight) were amended with 50 μg C g^−1^ dry soil of universally ^13^C-labeled substrate (5 at%, either glucose or vanillin) dissolved in deionized water, the amendment adjusting the soil samples to 60% of their water-holding capacity. The level of substrate addition approximated 10% of MBC of the bulk soil, representing a C-substrate addition within the natural soil range and therefore exerting minimal impact on microbial metabolism [57]. One set of samples received deionized water only to serve as controls. The samples were sealed inside 250-ml Mason jars and incubated at 25°C for 48 h. At the end of the incubation period, headspace gas samples (12 ml) were collected using a gas-tight syringe and injected into pre-evacuated exetainer vials. Total CO_2_ concentrations were measured by gas chromatography (Agilent 7890A gas chromatograph; Agilent Technologies, USA) and ^13^CO_2_ concentrations using isotope ratio mass spectrometry (IRMS, MAT253; Thermo Fisher Scientific, Waltham, MA, USA). Substrate incorporation into microbial biomass carbon was measured using ^13^C analysis of the extracts of fumigated and non-fumigated soils from the chloroform fumigation-extraction approach, using a TOC-VWP interfaced to a MAT253 IRMS. Microbial SUE was calculated using Eqs. (7)–(10) [52].


(7) \begin{eqnarray*} {\kern0em }^{13}\mathrm{MB}{\mathrm{C}}_x=\frac{{\kern0em }^{13}\mathrm{DO}{\mathrm{C}}_{\mathrm{F}}\times \mathrm{DO}{\mathrm{C}}_{\mathrm{F}}-{\kern0em }^{13}\mathrm{DO}{\mathrm{C}}_{\mathrm{NF}}\times \mathrm{DO}{\mathrm{C}}_{\mathrm{NF}}}{\mathrm{DO}{\mathrm{C}}_{\mathrm{F}}-\mathrm{DO}{\mathrm{C}}_{\mathrm{NF}}} \end{eqnarray*}

(8) \begin{eqnarray*} {\kern0em }^{13}\mathrm{MB}\mathrm{C}\%=\frac{{\kern0em }^{13}\mathrm{MB}{\mathrm{C}}_{\mathrm{t}}-{\kern0em }^{13}\mathrm{MB}{\mathrm{C}}_{\mathrm{c}}}{{\kern0em }^{13}\mathrm{sol}-{\kern0em }^{13}\mathrm{MB}{\mathrm{C}}_{\mathrm{c}}}\times 100 \end{eqnarray*}

(9) \begin{eqnarray*} {\kern0em }^{13}\mathrm{MBC}=\left(\mathrm{DO}{\mathrm{C}}_{\mathrm{F}}-\mathrm{DO}{\mathrm{C}}_{\mathrm{NF}}\right)\times \frac{{\kern0em }^{13}\mathrm{MBC}\%}{100} \end{eqnarray*}

(10) \begin{eqnarray*} \mathrm{Microbial}\ \mathrm{SUE}=\frac{{\kern0em }^{13}\mathrm{MBC}}{{\kern0em }^{13}\mathrm{MBC}+{\kern0em }^{13}R} \end{eqnarray*}

where ^13^DOC_F_, DOC_F_, and ^13^DOC_NF_ represent the at%^13^C and total C concentrations (μg C g^−1^ soil) of K_2_SO_4_ extracts of fumigated and non-fumigated soils, respectively. ^13^MBC*_t_* and ^13^MBC*_c_* (denoted as ^13^MBC*_x_*) are the at%^13^C of isotopically treated samples and natural abundance controls, and ^13^sol is the at%^13^C of the substrate solution. ^13^MBC relates to microbial growth (μg C g^−1^ soil), and ^13^*R* represents cumulative respiration derived from the labeled substrate during the 48-h incubation period.

By simultaneously determining the microbial utilization efficiency of both glucose and vanillin, relative anabolic efficiency (RAE) was calculated as


(11) \begin{eqnarray*} \mathrm{RAE}=\frac{{\mathrm{SUE}}_{\mathrm{Glc}}}{{\mathrm{SUE}}_{\mathrm{Van}}} \end{eqnarray*}

where SUE_Glc_ and SUE_Van_ represent the microbial utilization of glucose and vanillin, respectively. A larger RAE value suggests that microbial biomass production is more efficient on glucose-derived C than on vanillin-derived C, relative to substrate-derived respiratory C losses.

### Statistical analysis

All statistical analyses were performed with R (v4.5.2) following four steps. First, linear mixed-effects models were constructed using the *nlme* package. Management practices, warming, and their interaction were included as fixed effects, whereas plot (Block) was included as a random effect to account for the nested experimental design. These models were used to assess the overall and interactive effects of management and warming on microbial metabolic properties (CUE, respiration, and growth) and predictive variables including substrate availability and quality, microbial community traits (microbial community composition, *rrn* copy numbers, and copiotrophs and oligotrophs based on microbial functional traits), and edaphic conditions. Post hoc pairwise comparisons were then performed to evaluate the specific effects of warming within each management system separately, with *P* values adjusted using the Bonferroni correction. Second, microbial community responses were assessed. Alpha diversity (richness and Shannon index) was compared between treatments, whereas community composition (Beta diversity) was analyzed using Bray–Curtis dissimilarity via PERMANOVA, ANOSIM, and MRPP (*vegan* package). Differentially abundant ASVs were identified using ALDEx2 and ANCOM-BC. Third, warming-induced functional shifts were examined using metagenomic data. Differentially abundant CAZy families were identified via LEfSe, and functional dispersion (FDis) of C-degrading genes in MAGs was calculated using fundiversity package. Finally, piecewise structural equation modeling (pSEM) was applied as a hypothesis-guided exploratory path analysis to evaluate the direct and indirect associations among management, warming, substrate traits, microbial traits, and microbial C allocation. The pSEM was constructed using 16 independent field samples, corresponding to four biological replicates per treatment. To reduce model complexity relative to the available sample size, correlated variables were summarized into biologically interpretable composite indices using principal component analysis before inclusion in the pSEM. The pSEM was fitted using the R package *piecewiseSEM*, model fit was evaluated using Fisher’s *C* statistic, and standardized effects were estimated using 9999 bootstrap iterations with the R package *semEff*.

## Results

### Microbial C metabolic and substrate traits

**Figure 2:**
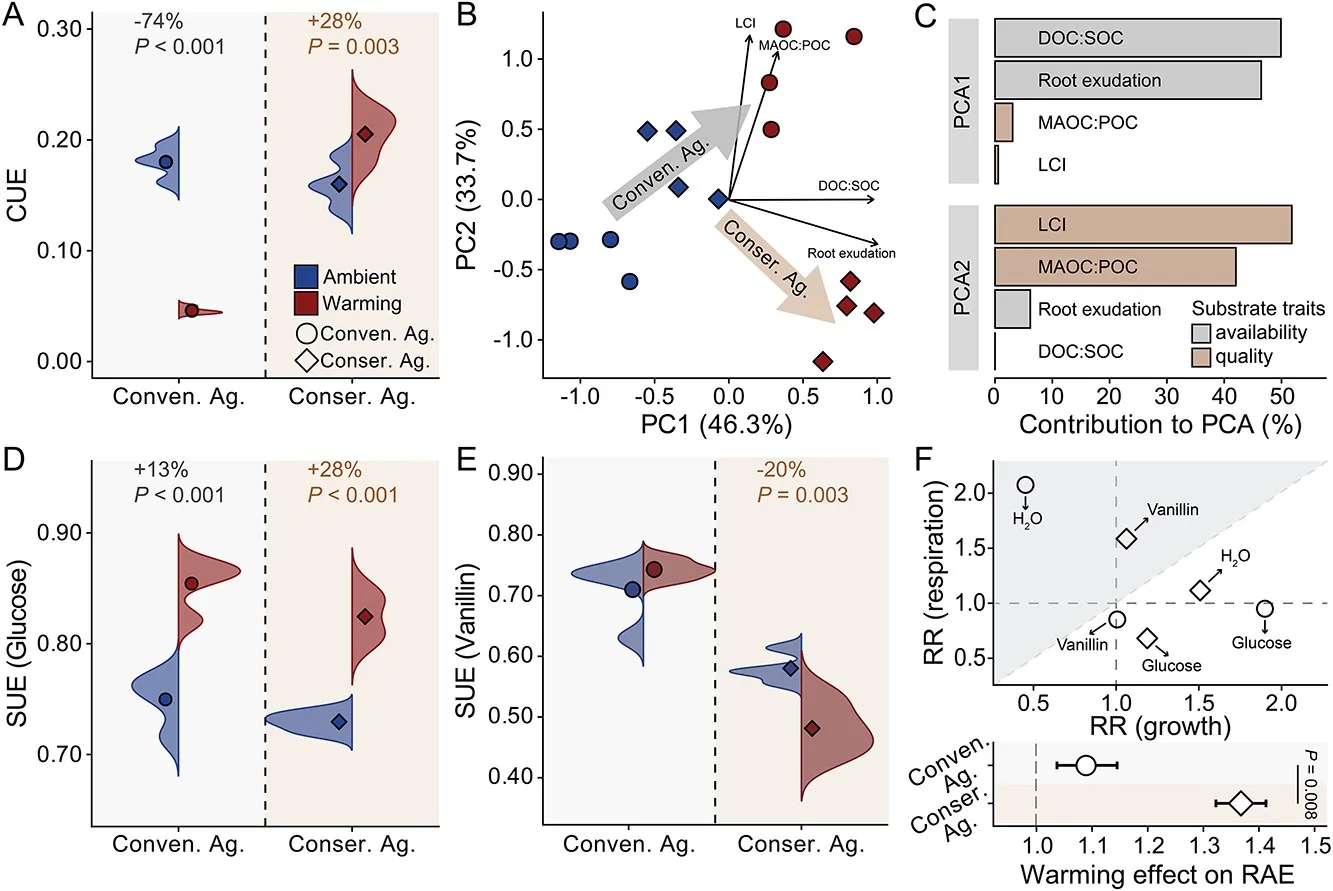
Effects of warming and management on microbial carbon and substrate use efficiencies and substrate traits. (**A**) Changes of microbial carbon use efficiency estimated by ^18^O-H_2_O approach (*n* = 4). (**B**–**C**) Principal component analysis (PCA) of substrate availability and quality under warming and different agricultural management systems. Relative contribution (% loading) of each variable to the principal components. Changes of microbial substrate use efficiency estimated by ^13^C-glucose in (**D**) and ^13^C-vanillin in (**E**) approach (*n* = 4). (**F**) Changes in microbial carbon allocation and relative anabolic efficiency caused by warming. Data are presented as violin plots with mean values (point; *n* = 4). The gray dotted line indicates that warming has no effect on microbial relative anabolic efficiency in (**F**). The effects of management, warming, and their interaction on the response variable were evaluated using linear mixed-effects models with plot as a random effect. Block was included as a random effect to account for experimental design structure. Fixed effects and interaction were evaluated by ANOVA, and *post hoc* pairwise comparisons of estimated marginal means were used to examine warming effects within each management system. CUE, carbon use efficiency; DOC, dissolved organic carbon; SOC, soil organic carbon; LCI, lignocellulose index; POC, particulate organic carbon; MAOC, mineral-associated organic carbon. RAE, microbial substrate relative anabolic efficiency. Conven. Ag., conventional agriculture; Conser. Ag., conservation agriculture. Data for root exudation carbon input (Oct 2019–May 2020 wheat growth) in panel (**B**) were adapted from a previously published dataset [28].

After 11 years of experimental warming (+2°C; Fig. S1), physiological measurements from the 2021 sampling confirmed that warming effects on microbial CUE remain strongly management dependent (*P* < .001; Table S3), consistent with previously reported decadal trends [26]. Specifically, warming increased CUE by 28% under conservation agriculture but decreased it by 74% under conventional management (Fig. 2A), reflecting contrasting microbial C allocation between growth and respiration. Under conventional agriculture, warming increased respiratory C loss but suppressed microbial growth, whereas under conservation agriculture warming stimulated microbial growth despite increased respiration (Fig. 2F and Fig.  S2). Because community-level CUE represents an emergent property of microbial metabolic trade-offs, we next examined whether these divergent responses were associated with warming-induced shifts in the biochemical and thermodynamic quality of soil substrates.

Principal component analysis showed that warming and management altered substrate availability and quality (Fig. 2B and C). Warming primarily separated substrate traits along PC1, which explained 46% of the variance and was largely associated with increased substrate availability across both management systems. Specifically, warming increased root exudation under both conservation agriculture (48%, *P* < .001) and conventional agriculture (37%, *P* = .002, Fig. S3). Similarly, DOC:SOC ratios increased with warming under both management regimes (*P* = .001 for conventional agriculture; *P* = .003 for conservation agriculture), indicating an overall enhancement of soil C availability (Fig. S3). In contrast, substrate quality separated along PC2 (33% of the variance) and responded differently to warming depending on management (Fig. 2B and C). Under conventional agriculture, warming reduced substrate quality, whereas under conservation agriculture it increased. These contrasting responses were reflected by opposing trends in the lignocellulose index (LCI) and MAOC:POC ratios. The enzymatic LCI increased under conventional agriculture (*P* = .004) but decreased under conservation agriculture (*P* = .001; Fig. S3; Table S3). Consistently, warming increased MAOC:POC ratios by 35% under conventional agriculture (*P* < .001; Fig. S3), whereas no significant effect was observed under conservation agriculture. Apart from these changes in substrate traits, other abiotic factors also showed management-dependent responses to warming. Soil pH was insensitive to warming in both management systems, whereas DOC and NO_3_^−^–N increased consistently under warming; total nitrogen increased only under conservation agriculture (Table S4).

We further applied ^13^C-glucose and ^13^C-vanillin as contrasting readily available carbohydrate and lignin-derived aromatic substrates to quantify substrate relative anabolic efficiency (RAE). Warming consistently enhanced glucose utilization efficiency (+28% under conservation agriculture and +13% under conventional agriculture; *P* < .001; Fig. 2D) by stimulating microbial growth while reducing respiratory carbon allocation (Fig. S2). In contrast, warming had no significant effect on vanillin utilization efficiency under conventional management but significantly reduced it under conservation agriculture (−20%; *P* = .003; Fig. 2E), primarily due to increased respiratory carbon losses (Fig. S2). Overall, warming increased microbial RAE, estimated as the ratio SUE_Glc_:SUE_Van_. Under conservation agriculture, RAE increased by 1.37-fold compared with a 1.09-fold increase under conventional management (*P* = .008; Fig. 2F). The magnitude of this shift was strongly management dependent, with RAE increasing by 36% under conservation agriculture but only by 9% under conventional management (*P* = .008; Fig. 2F). Regression analysis further showed that warming-induced changes in CUE were primarily associated with shifts in RAE (*R*^2^ = 0.73, *P* = .007; Fig. S4). Specifically, CUE responses were negatively correlated with vanillin utilization efficiency (*R*^2^ = 0.68, *P* = .010) but showed no relationship with glucose utilization efficiency.

### Microbial community composition, diversity, and life-history traits

**Figure 3:**
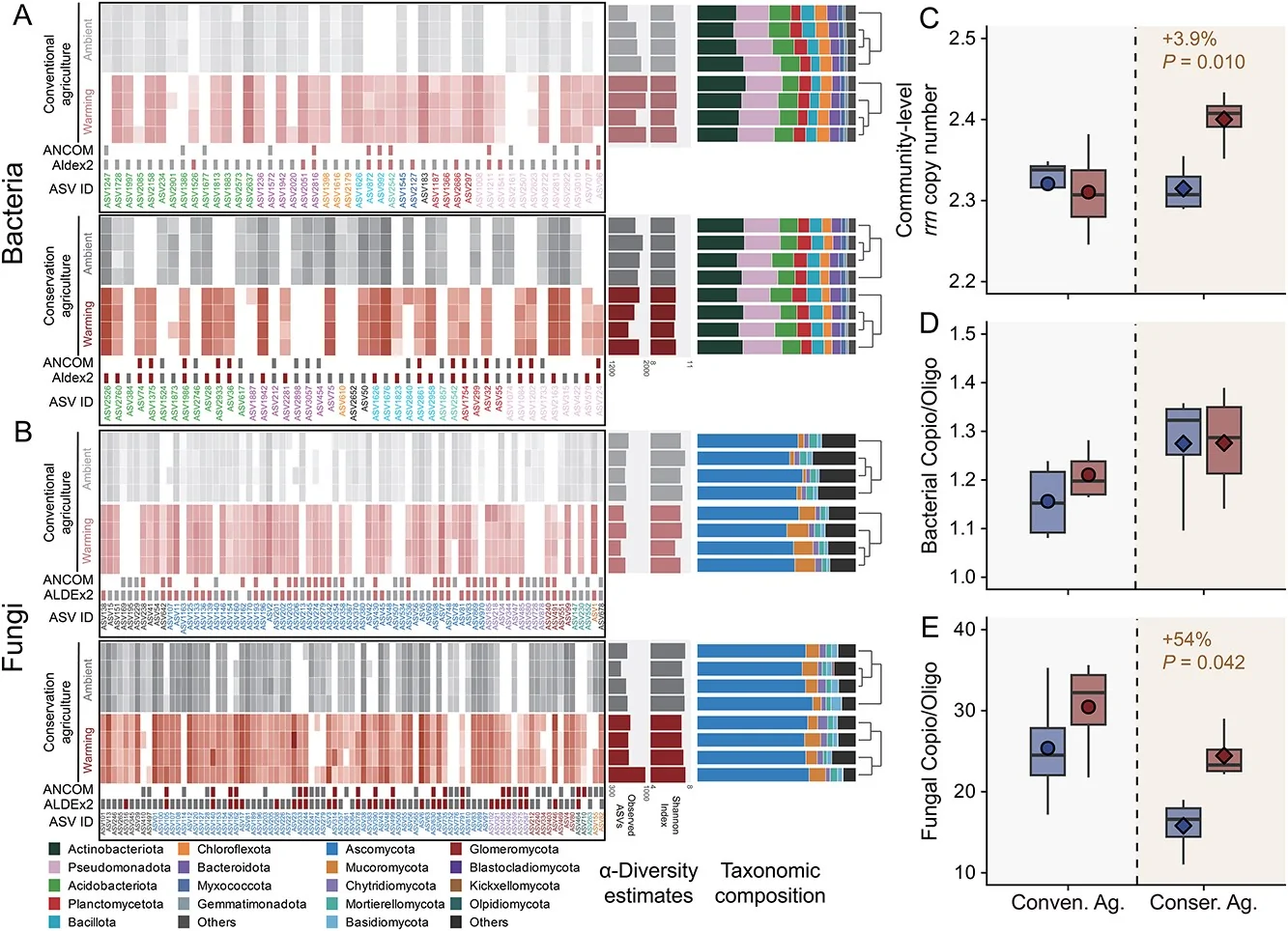
Effects of warming and management on microbial diversity, community structure, and life-history strategies. (**A**–**B**) Changes in microbial diversity and community composition of bacteria and fungi, respectively. (**C–E**) Changes in microbial life-history strategies estimated by community-level *rrn* copy numbers (**C**), bacterial copiotrophic/oligotrophic taxa ratios (**D**), and fungal copiotrophic/oligotrophic taxa ratios (**E**). Each vertical line in (**A**) and (**B**) represents a unique ASV, where color intensity indicates the log-normalized abundance, and no color indicates an ASV that was either not detected or was removed during prevalence filtering. The colored bars below indicate ASVs that were enriched in specific temperature treatments as determined by analysis of compositions of microbiomes with bias correction (ANCOM-BC) or ANOVA-like differential expression (Aldex2). Diversity estimates charts show the observed richness and the Shannon diversity index. The white dot in each box shows the average (*n* = 4). The ends of the boxes represent the 25th and 75th percentiles, and the whiskers refer to the 10th and 90th quartiles. The effects of management, warming, and their interaction on the response variable were evaluated using linear mixed-effects models with plot as a random effect. Block was included as a random effect to account for experimental design structure. Fixed effects and interaction were evaluated by ANOVA, and *post hoc* pairwise comparisons of estimated marginal means were used to examine warming effects within each management system. Copio/Oligo, the ratio of copiotrophic/oligotrophic taxa. Conven. Ag., conventional agriculture; Conser. Ag., conservation agriculture.

Long-term warming increased the F:B ratio under conservation agriculture (*P* = .028; Fig. S5) but had no effect under conventional agriculture. Warming did not affect the Shannon diversity of either soil bacteria or fungi (Fig. 3A and B; Fig. S6), yet it induced shifts in microbial community composition (Fig. 3A and B; Fig. S7–S8). Numerous taxa changed in relative abundance under warming, with responses varying between management systems (Fig. 3A and B). In warmed soils, the relative abundance of oligotrophic taxa within the phylum Chloroflexota decreased under conservation agriculture (*P* = .001; Fig. S7). For fungi, warming increased the relative abundance of Mucoromycota-affiliated taxa under conventional agriculture (*P* < .001; Fig. S8). Conversely, warming reduced the relative abundance of oligotrophic taxa within the phylum Basidiomycota under conservation agriculture (*P* < .001; Fig. S8).

**Figure 4:**
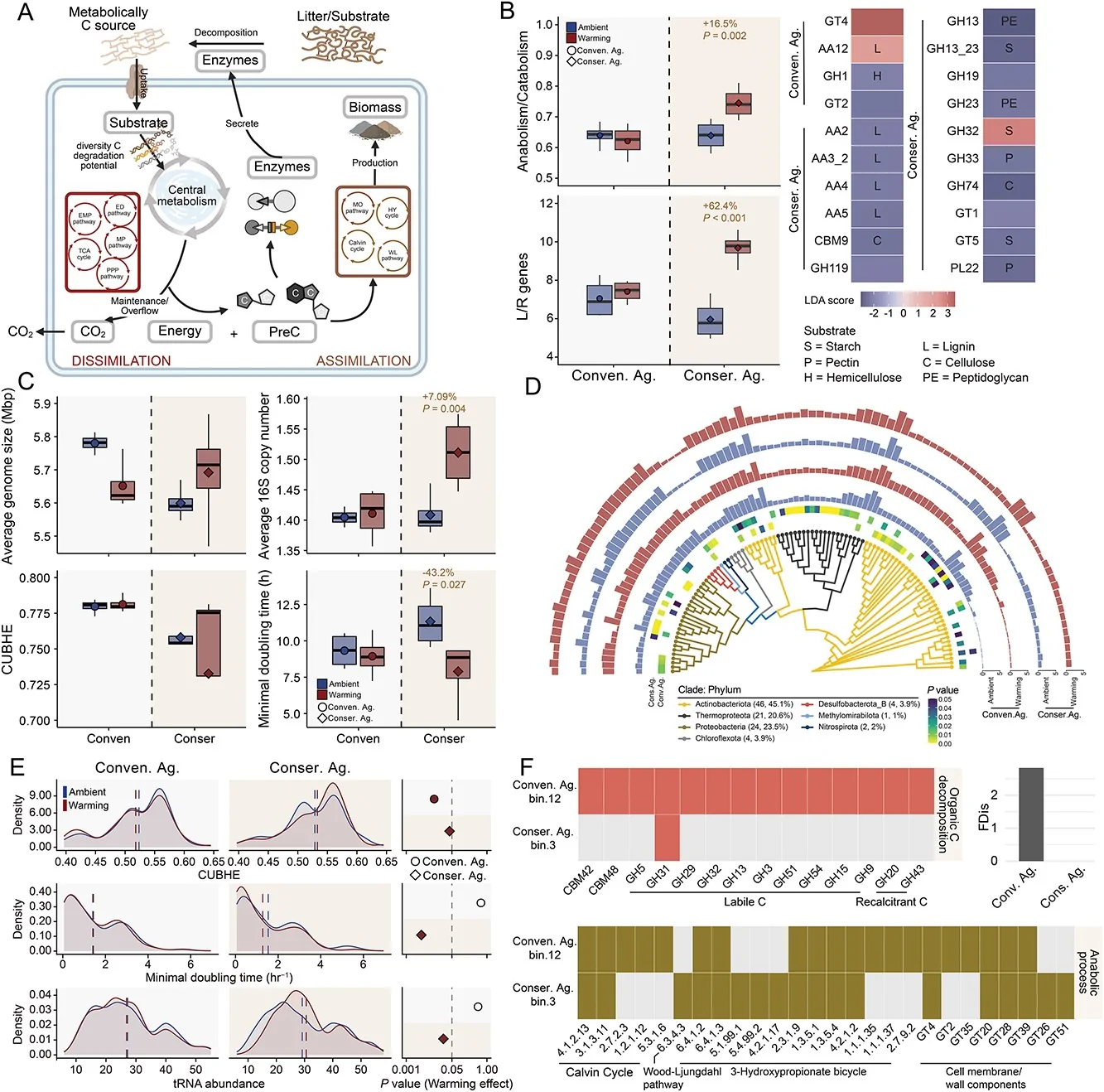
Effects of warming and management on microbial metabolism processes and metagenomic traits. (**A**) Schematic diagram of soil microbial C metabolism in agricultural ecosystems comprising microbial C catabolic and anabolic processes. Microbial anabolic processes include the Calvin cycle, 3-hydroxypropionate bi-cycle (3-HP cycle), and Wood–Ljungdahl pathway (WL) [73, 74]. (**B**) Ratios of gene abundances under ambient and warming conditions in both agricultural management systems, including the anabolic/catabolic gene abundance ratio and the labile/recalcitrant C degradation gene abundance ratio (L/R genes). Anabolic/catabolic gene abundance ratios based on KEGG database annotations (Table S8). Boxes represent the interquartile range for ambient and warming treatments. The dot in each box shows the average (*n* = 4). The effects of management, warming, and their interaction on the response variable were evaluated using linear mixed-effects models with plot as a random effect. Block was included as a random effect to account for experimental design structure. Fixed effects and interaction were evaluated by ANOVA, and *post hoc* pairwise comparisons of estimated marginal means were used to examine warming effects within each management system. Heatmap showing the linear discriminant analysis (LDA) scores computed for carbohydrate-active enzyme gene families being differentially abundant (LDA score > 2, unadjusted *P* < .05) between warming and control conditions under both management systems. CBM, carbohydrate-binding module; GH, glycoside hydrolase; GT, glycosyltransferase. (**C**) Responses of genomic traits at the read and contig levels to warming under both agricultural management systems. (**D**) Differences in the abundance of metagenome-assembled genomes (MAGs) across all treatments. The heatmap illustrates the *P* values of abundance, determined by *t*-tests, for MAGs under warming and ambient conditions under both management systems. (**E**) Responses of genomic traits at MAG levels to warming under both agricultural management systems. (**F**) MAGs exhibiting the most pronounced abundance differences between warming and ambient conditions consistently reached peak abundance levels under elevated temperatures across both management systems. Height of the bar indicates MAG abundance. A Presence–absence plot shows the profiles of selected genes in MAGs (Conven. Ag. bin.12 and Conser. Ag. bin.3). FDis, functional dispersion; Conven. Ag., conventional agriculture; Conser. Ag., conservation agriculture. Panel (**A**) was created with BioRender. Ling, J. (2026) https://BioRender.com/wt8777q.

Although warming had no effect on bacterial and fungal Shannon diversity, β-dispersion (“distance to centroid”) analyses revealed that warming increased the dissimilarity of bacterial and fungal communities under conservation agriculture (Fig. S9). These changes resulted in significant dissimilarity of fungal communities between warming and ambient conditions in conservation agriculture (Table S5). Warming also increased community-level *rrn* copy number by 3.8% under conservation agriculture (*P* = .010; Fig. 3C). Under both management systems, warming did not affect bacterial copiotrophic-to-oligotrophic ratios (Fig. 3D); however, warming increased fungal copiotrophic-to-oligotrophic ratios by 54% under conservation agriculture (*P* = .042; Fig. 3E). Under conservation agriculture, warmed soils had a higher average 16S rRNA gene copy number than ambient soils (+7.0%, *P* = .004; Fig. 4C), whereas predicted minimal doubling time decreased by 43% (*P* = .027; Fig. 4C). By contrast, these genomic traits showed no significant warming response under conventional agriculture. We further examined life-history traits at the MAG level, weighted by their community abundance. We observed a divergent response of the codon usage bias for highly expressed genes (CUBHE) to soil warming: warming significantly decreased CUBHE under conventional agriculture (*P* = .010) but increased it under conservation agriculture (*P* = .040; Fig. 4E). This shift under conservation agriculture was accompanied by a significant reduction in the minimal doubling time (*P* = .003) and a concomitant increase in tRNA abundance (*P* = .024; Fig. 4E).

### Specialized anabolism and catabolism profiles of microbiomes

The allocation of C between microbial anabolic and catabolic pathways determines CUE (Fig. 4A). Under conservation agriculture, rising temperatures increased the anabolic-to-catabolic ratio, indicating a microbial shift toward growth-oriented processes (*P* = .002; Fig. 4B). Given the contrasting substrate relative anabolic efficiency of microbial communities between the two management systems, we further examined warming-induced changes in the microbial genetic potential for C resource acquisition. Specifically, QMEC analysis revealed that warming increased the abundance of labile-to-recalcitrant (L/R) C acquisition genes under conservation agriculture (*P* < .001; Fig. 4B and Fig. S10). Consistently, metagenomic analysis showed an enrichment of genes associated with labile-C degradation and a concurrent depletion of genes involved in recalcitrant C degradation (Fig. 4B and Fig. S11).

Of the 106 medium-quality MAGs generated from 16 metagenomes after dereplication and quality filtering, 46 MAGs were assigned to Actinomycetota, 25 to Thermoproteota, and 24 to Pseudomonadota (Fig. 4D). Among these, two MAGs—TW.bin.12 (Thermoproteota, from conventional agriculture) and NW.bin.3 (Actinomycetota, from conservation agriculture)—showed the strongest increases in abundance under warming compared to ambient conditions (Fig. 4D). Detailed genomic analyses of these two MAGs revealed distinct differences in the encoded metabolic potential for organic C decomposition and catabolism. TW.bin.12 encoded a broader array of C-degradation genes, whereas NW.bin.3 contained only genes for labile-C degradation but exhibited a greater diversity of genes associated with anabolic pathways (Fig. 4F).

### Controls on microbial C metabolic traits

**Figure 5:**
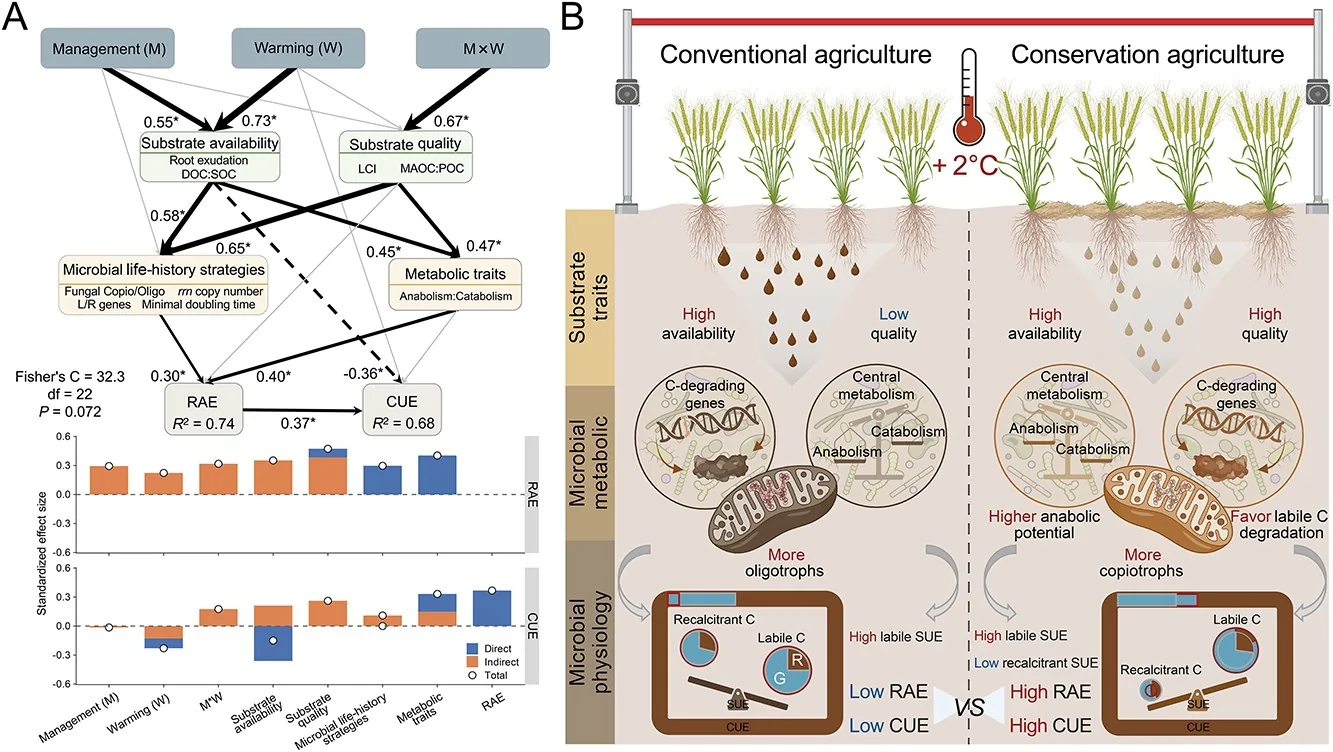
Pathways regulating the warming response of microbial carbon use efficiency and substrate relative anabolic efficiency. (**A**) Piecewise structural equation model analysis (pSEM) showing the direct and indirect effects of biotic and abiotic factors on CUE and RAE. PCA was applied to derive multivariate indices for substrate availability, substrate quality, and microbial life-history strategies. Substrate availability (based on root exudation and the dissolved organic carbon/soil organic carbon ratio) and quality (based on the mineral-associated organic carbon/particulate organic carbon ratio and lignocellulose index) variables were jointly analyzed in one PCA, with first principal component axis scores of PCA representing substrate availability and second principal component axis scores of PCA representing substrate quality (Fig. 2B and C). Microbial life-history strategies (based on fungal copiotrophic/oligotrophic taxa, weighted average rRNA operon copy number of the bacterial community, the labile/recalcitrant carbon degradation gene ratio, and the reciprocal of minimal doubling time) were analyzed in a separate PCA, and first principal component axis scores of PCA were used as a composite microbial trait index (Fig. S13). The catabolic/anabolic gene abundance ratio represents metabolic traits. Arrow thickness represents the strength of the relationships. Solid and dotted black arrows represent significant positive and negative relationships (*P* < .05), respectively; while the remaining arrows represent nonsignificant pathways. Numbers adjacent to the arrows represent standardized path coefficients. Percentages close to the target variables indicate the variance explained by the model (*R*^2^). The pSEM was constructed using 16 independent field samples, with four biological replicates per treatment. Each biological replicate corresponded to one independent field plot. The goodness-of-fit statistics for the model were Fisher’s *C* = 32.3, *d.f.* = 22, and *P* = .072. Standardized effects were estimated using 9999 bootstrap iterations. ^*^.01 < *P* < .05, ^**^.001 < *P* < .01, ^***^P < .001. (B) Conceptual diagram showing the interactive effects of cropping system management and warming on CUE and RAE and the driving mechanisms. In conventional agriculture, warming promoted the supply of larger quantities of more readily available but lower-quality C substrates, shifting microbial communities toward more oligotrophic taxa. These lower-quality inputs suppressed microbial CUE and caused a relative increase in the utilization of recalcitrant C, thereby reducing microbial relative anabolic efficiency for the utilization of labile substrates. By contrast, under conservation agriculture, warming increased both substrate availability and substrate quality. This is reflected in microbial traits, such as an increased propensity for labile-C decomposition and anabolic processes, which together promoted CUE, the efficiency in the utilization of labile C, and RAE. Catabolic pathways (Catab.) are those that depolymerize and oxidize complex organic substrates to generate metabolic energy. They include Embden–Meyerhof–Parnas glycolysis (EMP), Entner–Doudoroff pathway (ED), tricarboxylic acid cycle (TCA), pentose phosphate pathway (PPP), and other minor catabolic routes indicated as methanogenesis pathway (MP). Anabolic (Anab.) processes are pathways that assimilate C and other precursors to build biomass and biosynthetic intermediates. They include Calvin cycle (CC), the Wood–Ljungdahl pathway (WL), 3-hydroxypropionate bi-cycle (HY), and methane oxidation (MO). In microbial physiology, the outline corresponding to the ambient and elevated-temperature treatments indicate microbial growth and respiration under the respective temperature conditions. The shaded areas denote the relative contributions of microbial growth and microbial respiration to total microbial carbon flux. CUE, carbon use efficiency. RAE, relative anabolic efficiency. Panel (**B**) was created with BioRender. Ling, J. (2026) https://BioRender.com/y8zj1jq.

To disentangle the hierarchical mechanisms regulating microbial CUE and RAE, we conducted pathway analysis using piecewise structural equation modeling (pSEM; Fig. 5A and Table S6). The model captured a substantial proportion of the variance for both RAE (*R*^2^ = 0.74) and CUE (*R*^2^ = 0.68). The pathway analysis identified RAE as a primary proximal driver of CUE, exerting a strong positive direct effect, whereas substrate availability acted as a direct negative constraint. Upstream of this regulation, RAE was predominantly governed by microbial life-history strategies and metabolic traits. These microbial functional traits were, in turn, distinctly shaped by environmental and anthropogenic filters. Specifically, management and warming indirectly affected RAE primarily through changes in substrate availability. Increased substrate availability was associated with shifts in microbial life-history strategies and metabolic traits, which together contributed to variation in RAE. Concurrently, the interactive effect of management and warming (M × W) significantly altered substrate quality, which indirectly cascaded to RAE via shifts in metabolic traits and microbial life-history strategies. Collectively, this framework elucidates a mechanistic cascade where global change stressors and agricultural practices reconfigure substrate traits, microbial life-history strategies, and metabolic traits, thereby shifting microbial substrate relative anabolic efficiency to ultimately dictate community-level CUE.

## Discussion

Adopting conservation agriculture in arable farmlands is widely advocated to address global food security and climate change challenges through the ecosystem benefits associated with SOC accrual [25, 58, 59]. The capacity of conservation agriculture to deliver such ecosystem services, including C sequestration and sustainable food production, ultimately depends on the metabolic responses of soil microorganisms to global warming. By combining a long-term field experiment with complementary laboratory incubations (Fig. 1), we demonstrate that warming increased microbial CUE when growing on native SOC (assessed by substrate-independent H_2_^18^O labeling) but reduced microbial SUE of vanillin-derived C (determined using substrate-dependent ^13^C labeling) in our long-term agricultural experiment (Fig. 2). Building on these findings, we propose a conceptual framework to illustrate the potential mechanisms underlying the response of microbial physiological metabolism to prolonged warming under conventional and conservation agriculture (Fig. 5).

Understanding of the interactive effects of long-term warming and management practices on microbial CUE remains limited, despite previous investigations examining the dynamic metabolic responses of soil microorganisms to warming [60–62]. In our study, chronic soil warming produced contrasting patterns of microbial CUE depending on the crop management system: CUE decreased under conventional agriculture but increased under conservation agriculture (Fig. 2A). This finding challenges the prevailing notion that microbial CUE generally declines with rising soil temperature [60, 63]. Using a substrate-independent ^18^O-H_2_O labeling approach, we confirmed that the effect of warming on CUE was contingent on the management system (Table S3). Contrary to the common assumption that warming constrains microbial growth by elevating the energetic costs of maintaining existing biomass [64], we observed that microbial growth was stimulated to a greater extent than respiration when substrate availability was sufficient (Fig. 2F).

The positive response of microbial CUE to warming under conservation agriculture likely arises from complex crop–soil–microorganism interactions. First, 11-year field warming under conservation agriculture increased crop yield and plant C inputs [26, 37]. The combination of enhanced root exudation (Fig. S3), elevated DOC:SOC (Fig. S3), reduced LCI (Fig. S3), and unaltered MAOC:POC ratios (Fig. S3) collectively improved soil C availability and C quality, thereby promoting microbial CUE (Fig. 5A). This higher input of plant-derived carbon under warming likely helped mitigate the negative effects of temperature on microbial CUE and contributed to maintaining SOC under conservation agriculture. These findings corroborate our first hypothesis, indicating that long-term warming under conservation agriculture enhances both the supply and quality of plant-derived C inputs, thereby facilitating more efficient microbial C utilization. Second, an increased F:B ratio could have contributed to higher microbial CUE in conservation agriculture under warming conditions (Fig. S5), reflecting a shift in microbial C metabolism and functional potential. This aligns with previous research showing that CUE tends to be higher in fungal-dominated than in bacterial-dominated soil communities [65, 66]. Third, soils under conventional agriculture were drier than those under conservation agriculture and warming further reduced soil moisture in both systems (Fig. S1). Reduced soil moisture is known to directly constrain microbial biomass and growth by limiting the availability of dissolved substrates [67]. However, MBC increased under conservation agriculture despite warming (Fig. S12), suggesting greater allocation of resources to anabolic processes, which is consistent with the observed increase in CUE in this management system.

Because substrate availability increased in both management systems (Fig. S3), the observed divergence is unlikely to result solely from differences in C supply. We further considered soil pH because it is a key determinant of microbial community composition and metabolic activity [68]. However, pH did not differ significantly among treatments and was not significantly related to microbial growth, respiration, CUE, or substrate-specific SUE (Table S4 and Fig. S14), suggesting that pH shifts were unlikely to be the primary driver of these contrasting responses. Instead, our SEM suggested that microbial communities in the two management systems adopt distinct metabolic strategies in response to warming. The concurrent increase in CUE under conservation agriculture and its decline under conventional agriculture (Fig. 2A) supports this interpretation, pointing to a shift toward greater anabolic investment (Fig. 4B) and microbial growth (Fig. 2F) under conservation agriculture. Together, these results imply that changes in substrate quality altered microbial traits and thereby shaped the response of CUE to long-term warming (Fig. 5A).

Crop residue retention is a defining characteristic of conservation agriculture, enhancing SOC and protecting the soil surface from environmental stresses [69, 70]. In this study, chopped straw residues were retained on the soil surface under conservation agriculture, whereas residues were removed under conventional agriculture. Crop straw is composed of plant cell wall polymers with distinct chemical properties: cellulose consists of chains of glucose monomers, whereas lignin is composed of phenolic monomers. Most aerobic soil microorganisms possess metabolic pathways for glucose mineralization. In contrast, the oxidation of aromatic compounds derived from lignin—such as vanillin, a methoxylated phenolic aldehyde—is restricted to a comparatively small group of microorganisms [71]. To examine specific aspects of microbial metabolism and assess short-term microbial substrate relative anabolic efficiency, we used two representative monomers derived from crop residue decomposition: glucose (high bioavailability) and vanillin (low bioavailability; Fig. 1). Using those in a substrate-dependent ^13^C utilization approach, we found that long-term warming effects on substrate availability shaped both microbial community composition and SUE, thereby contributing to the distinct patterns of relative anabolic efficiency observed across management systems (Fig. 2F and Fig. 5).

Consistent with our first hypothesis, 11 years of field warming increased the utilization efficiency of ^13^C-glucose in both conventional and conservation agriculture, primarily due to greater microbial allocation to growth on this substrate (Fig. 2D and F). Glucose addition alleviates microbial C limitation in organic matter–poor but nutrient-rich agricultural soils and activates the anabolic machinery of dormant microorganisms with inherently high CUE. In contrast, negative or negligible CUE responses to soil temperature following glucose amendment have been reported in organic matter–rich but nutrient-poor forest soils [56, 72]. Unlike glucose, the substrate-specific use efficiency of added ^13^C-labeled vanillin responded differently to warming under the two management systems. Warming had little effect on the utilization efficiency of vanillin under conventional agriculture, but reduced it by 20% under conservation agriculture (Fig. 2E). This finding contradicts our third hypothesis, which proposed that warming would enhance microbial utilization efficiency of recalcitrant C through labile substrate depletion and microbial adaptation. In this 11-year field experiment, we found no clear evidence of substrate depletion under either management system (Fig. S3).

Instead, the dominant mechanism was microbial metabolic adaptation, particularly pronounced under conservation agriculture. Microbial communities shifted toward taxa and functional gene repertoires favoring the processing of labile C—evident from the dominance of copiotrophic taxa and a reduction in genes associated with recalcitrant C degradation (Fig. 3E and Fig. 4B). By contrast, the microbial community under conventional agriculture was dominated by oligotrophs adapted to the relatively greater availability of recalcitrant substrates, thereby supporting the efficient utilization of ^13^C-vanillin. The community-level shifts under warming were accompanied by corresponding functional genomic changes, with a restructuring of C-degradation gene profiles (Fig. 4B). The selective suppression of lignin-degrading genes under conservation agriculture implies a potential slowdown in the turnover of recalcitrant C, potentially contributing to the accrual of stable SOC pools. These responses were strongly management dependent, supporting our second hypothesis that warming-induced changes in microbial CUE are modulated by management practices through alterations in resource availability and soil conditions (Fig. 5). In summary, long-term warming interacted with management practices to alter substrate availability and quality, thereby reshaping microbial community composition and function and ultimately influencing microbial relative anabolic efficiency.

## Conclusions

Agricultural management modulates the microbial efficiency of organic matter utilization in response to warming by altering substrate availability and quality, and by shaping community-level functional traits. Conservation agriculture enhances the relative anabolic efficiency of labile-C sources and promotes microbial anabolic allocation and CUE, whereas conventional agriculture constrains microbial growth responses and limits CUE under prolonged warming. Warming-induced shifts toward copiotrophic microbial communities under conservation agriculture reduced the genetic potential for recalcitrant C degradation, thereby promoting the accumulation of stable soil C pools. These findings underscore the pivotal role of trait-based microbial resource allocation in regulating soil C cycling under climate change and emphasize the importance of integrating microbial functional ecology into the development of climate-smart land management strategies.

## Supplementary Material

Supplement_materials_wrag224

## Data Availability

The high-throughput dataset and metagenomic sequencing dataset are available in the NCBI SRA database with PRJNA923452, PRJNA923455, and PRJNA1392159. The processed data are archived on figshare (10.6084/m9.figshare.32494320).

## Code availability

All scripts for data processing and visualization are available via GitHub at https://github.com/ling9802/articles_code_backup/tree/main/management_warming_CUE.
