# Supplementary material for: Conservation agriculture buffers persistent carbon loss under warming by microbial adaptation
Source: ISME J. 2026 Aug 30;20(1):wrag224. doi: 10.1093/ismejo/wrag224 (PMC13584212; doi:10.1093/ismejo/wrag224)
Supplement: Supplementary file 1 [file supplement_materials_wrag224.pdf]

Supplementary Information

Title: Conservation agriculture buffers persistent carbon loss under warming by microbial adaptation

Running title: Microbial adaptation buffers C loss

Authors:

Jun Ling[1,2], Wolfgang Wanek[3], Kyle Mason-Jones[4,5], Jennifer A. J. Dungait[6,7], Luiz A. Domeignoz-Horta[8,9], Ruixing Hou[1,*], Yakov Kuzyakov[10], Fusuo Zhang[2], Jing Tian[2,*]

Institutional Affiliations:

1 Key Laboratory of Ecosystem Network Observation and Modeling, Institute of Geographic Sciences and Natural Resources Research, Chinese Academy of Sciences, 100101 Beijing, China.

2 State Key Laboratory of Nutrient Use and Management, College of Resources and Environmental Sciences, China Agricultural University, 100193 Beijing, PR China.

3 Division of Terrestrial Ecosystem Research, Department of Microbiology and Ecosystem Science, Center of Microbiology and Environmental Systems Science, University of Vienna, 1010 Vienna, Austria.

4 Soil Microbial Interactions, Department of Geoscience, University of Tübingen, 72076 Tübingen, Germany.

5 Cluster of Excellence (EXC 3121): TERRA – Terrestrial Geo-Biosphere Interactions in a Changing World, University of Tübingen, 72076 Tübingen, Germany.

6 College of Life and Environmental Sciences, University of Exeter, EX4 4QJ Exeter, UK.

7 Carbon Management Centre, Scotland's Rural College (SRUC), EH9 3JG Edinburgh, UK.

8 Swiss Federal Institute for Forest, Snow and Landscape Research WSL, 8903, Birmensdorf, Switzerland.

9 Université Paris-Saclay, INRAE, AgroParisTech, UMR EcoSys, 91120 Palaiseau, France.

10 Department of Soil Science of Temperate Ecosystems, Department of Agricultural Soil Science, University of Göttingen, 37077 Göttingen, Germany.

* Corresponding author

Email: tianj@igsnrr.ac.cn; tianj@cau.edu.cn, hourx@igsnrr.ac.cn

This PDF file includes:

- Supporting Notes 1 to 2
- Supplementary Figures 1 to 14
- Supplementary Tables 1 to 8
- SI References

Supplementary Note 1. Measurements of soil enzyme activity

We analyzed the activities of β-1,4-glucosidase and polyphenol oxidase. Soil suspensions were prepared by homogenizing 1 g soil (fresh weight) with 50 mM tris(hydroxymethyl)aminomethane (Tris) buffer using a vortex shaker for 1 min. The pH of the Tris buffer used for soil suspension was adjusted to 8.1, which is the mean pH of soils from the study region. The activity of BG as a C-targeting hydrolytic enzyme was assayed fluorometrically using black 96-well microplates, by mixing 50 μL soil suspension with 200 μL substrate (200 μM 4-methylumbelliferyl β-D-glucopyranoside). Microplates were incubated in the dark at 20 °C for 4 h. At the end of the incubation, 10 μL 1.0 M NaOH was added to stop the reaction. Fluorescence was measured using excitation at 360 nm and emission at 460 nm with an automated fluorometric plate reader (SYNERGY H1, Maryland, USA). Potential polyphenol oxidase (PPO) was measured spectrophotometrically using clear 96-well microplates by mixing 50 μL soil suspension, 100 μL Tris buffer, and solution containing 25 mM L-3,4-dihydroxyphenylalanine (L-DOPA). Absorbance was assayed at 450 nm using a microplate spectrophotometer (SYNERGY H1, Maryland, USA).

Supplementary Note 2. Bioinformatic analyses of metagenomic data

Metagenomic libraries were constructed from all samples and sequenced on the NovaSeq System (Shanghai Majorbio Biotechnology Co., Ltd., Shanghai, China). For sequence processing, Trimmomatic (version 0.39) was employed to remove adapters and trim the raw reads. The filtered clean reads from each sample were first assembled using MEGAHIT (version 1.1.2) [1]. Contigs with a length ≥ 300 bp were selected as the final assembling result, and the contigs were used for further gene prediction and annotation. Open reading frames (ORFs) in each assembled contig were predicted using Prodigal (version 2.6.3) [2]. ORFs were clustered and dereplicated at 95% sequence identity using MMseqs2 (v6.f5a1c) [3], resulting in a non-redundant gene catalog. To investigate shifts in microbial metabolic capabilities under warming and different agricultural management practices, predicted genes were annotated against various databases. Substrate utilization of carbohydrate-active enzymes (CAZymes) was inferred from metagenomic data based on functional annotations in the CAZy database and supported by relevant literature (Table S7). Additionally, genes were annotated against the KEGG database using Diamond (version 0.8.35) [4]. KEGG Orthology (KO) terms related to substrate utilization pathways were obtained from the KEGG database and published studies. Counts of predicted genes were normalized to transcripts per million (TPM). As a complementary approach, specific functional genes related to C cycling were quantified using the Quantitative Microbial Elemental Cycling (QMEC) method (a high-throughput quantitative PCR technique) to assess the microbial functional potential [5]. Fourteen functional genes involved in the degradation of starch (*amyA*, *amyX*, *apu* and *sga*), hemicellulose (*abfA*, *manB* and *xylA*), cellulose (*cdh* and *cex*), chitin (*chiA*), pectin (*pgu*), and lignin (*glx*, *lig* and *mpn*) were quantified using the QMEC method [5]. Copy numbers of relevant genes were calculated, and the absolute abundance of genes was calculated using the 16S rRNA gene as a reference. Considering that QMEC enables absolute quantification of functional genes via high-throughput qPCR, providing higher accuracy than the relative abundance estimates

obtained from shotgun metagenomic sequencing, we used QMEC data to quantify functional genes. Specifically, we designated the sum of the genes involved in oxidation of lignin as recalcitrant C degradation genes, while the sum of the genes involved in starch, hemicellulose, cellulose, pectin and chitin hydrolysis as labile C degradation genes. We classified the metabolic genes according to EC (Enzyme Commission) numbers obtained from KEGG annotations into catabolic and anabolic pathways, based on previous studies, and further grouped them into nine metabolic pathways (Table S8).

We performed genome binning with CONCOCT, MaxBin2, and MetaBAT2 within the MetaWRAP v1.3.2 framework [6]. We then consolidated and refined the resulting bins with the DAS tool and evaluated the completeness and contamination of metagenome-assembled genomes (MAGs) using CheckM [7]. Taxonomic classification was assigned using the Genome Taxonomy Database (R232). After dereplicating across all samples, we retained 106 medium-quality MAGs (completeness > 50%; contamination < 10%) for downstream analyses. To quantify MAG abundances, we cross-mapped sample reads to the dereplicated genome set using Bowtie 2 [8]. Finally, we functionally annotated the MAGs by identifying carbohydrate-active enzymes and mapping genes to KEGG pathways.

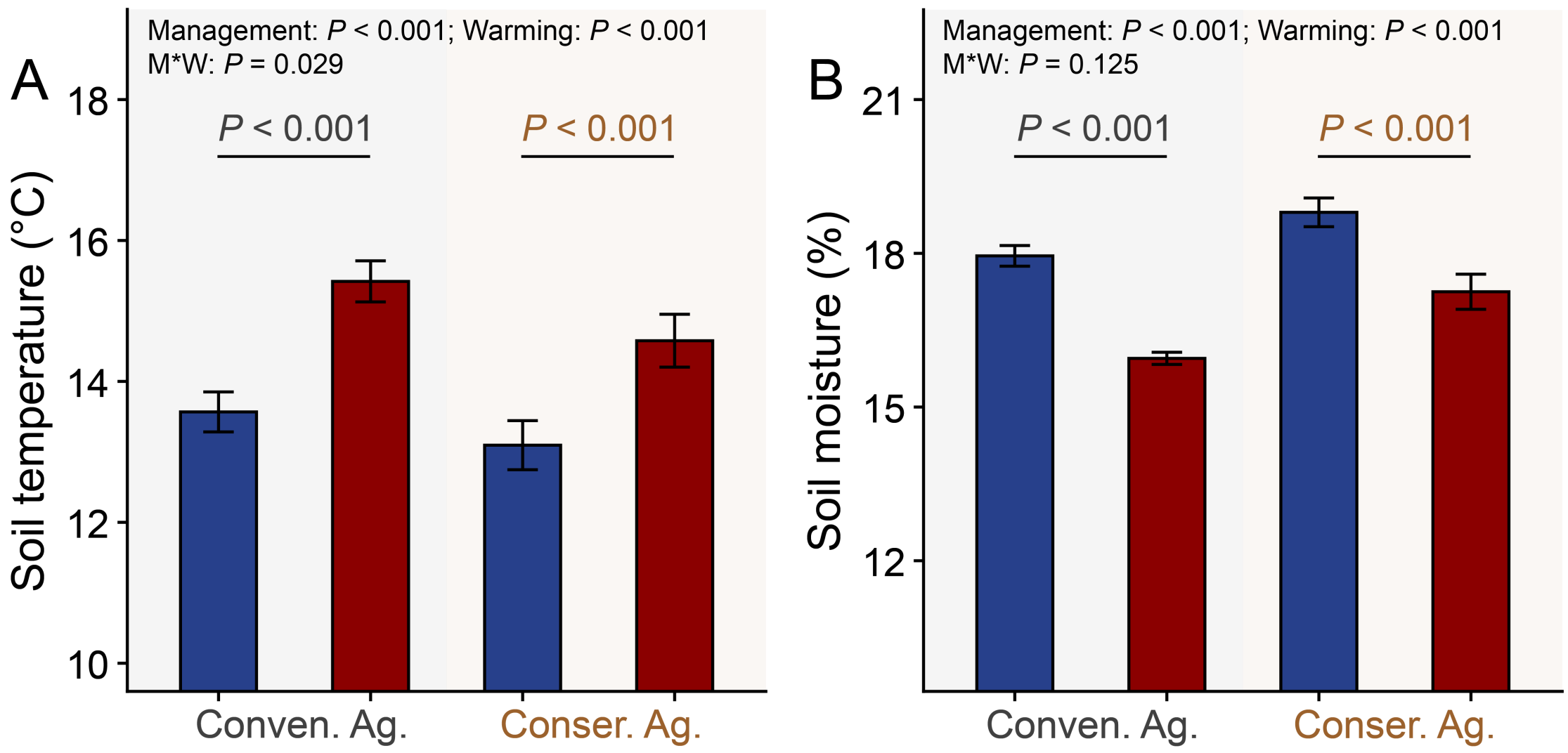

Figure S1. Effects of warming and management on annual averages of soil temperature and moisture. Data are presented as mean values ± s.e.m. ($n = 4$). The effects of management, warming, and their interaction on the response variable were evaluated using linear mixed-effect models with plot as a random effect. Block was included as a random effect to account for experimental design structure. Fixed effects and interaction were evaluated by ANOVA, and post-hoc pairwise comparisons of estimated marginal means were used to examine warming effects within each management system. Soil moisture (gravimetric, w/w, %). Conven. Ag., conventional agriculture; Conser. Ag., conservation agriculture.

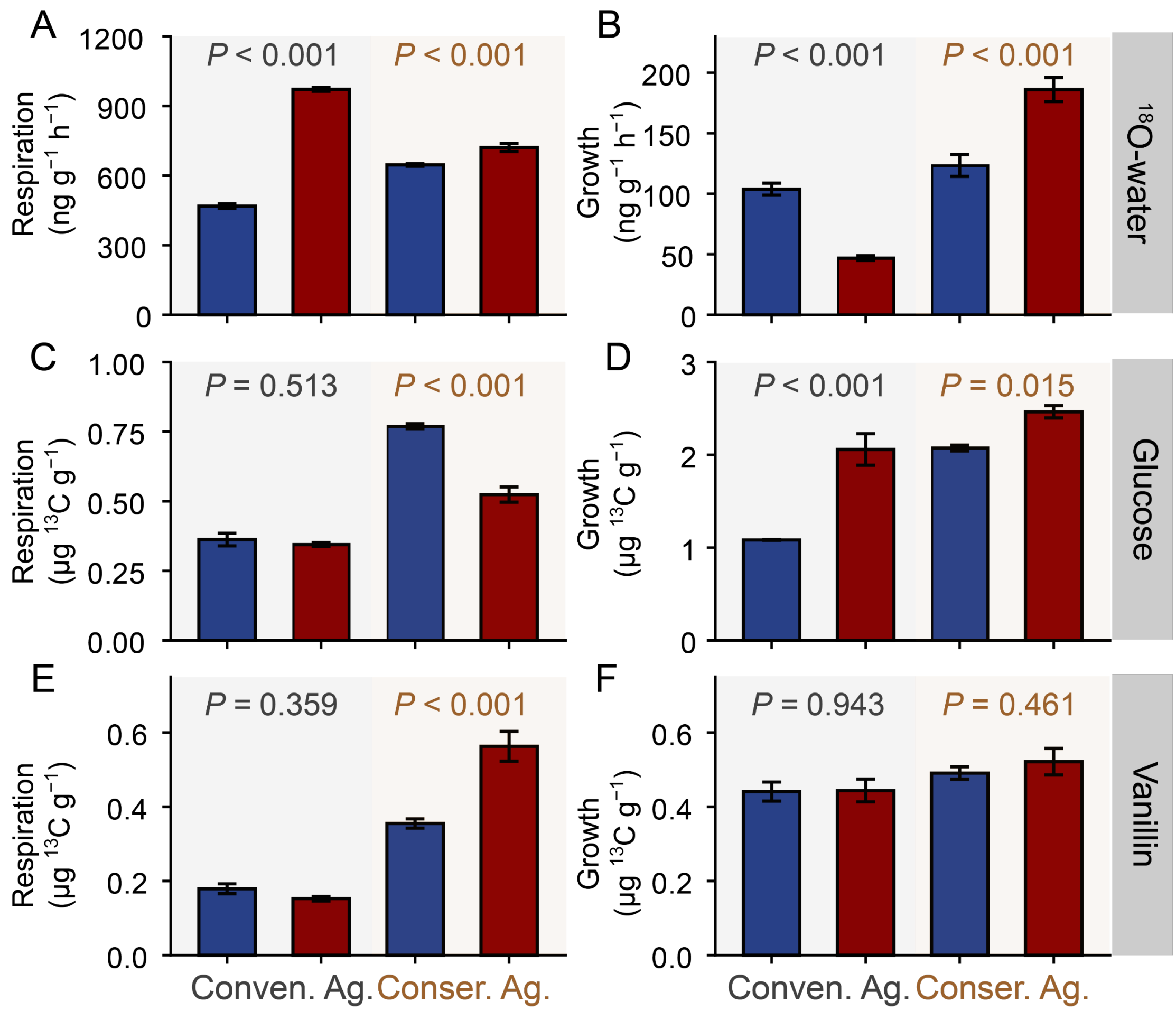

Figure S2. Effects of warming and management on microbial respiration and growth associated with microbial CUE and substrate-specific CUE under glucose and vanillin additions. Panels A–B show respiration and growth used to calculate microbial carbon use efficiency (CUE). Panels C–D show substrate-specific respiration and growth under glucose addition, and panels E–F show substrate-specific respiration and growth under vanillin addition. Data are presented as mean values ± s.e.m. ($n = 4$). The effects of management, warming, and their interaction on the response variable were evaluated using linear mixed-effect models with plot as a random effect. Block was included as a random effect to account for experimental design structure. Fixed effects and interaction were evaluated by ANOVA, and post-hoc pairwise comparisons of estimated marginal means were used to examine warming effects within each management system. Conven. Ag., conventional agriculture; Conser. Ag., conservation agriculture.

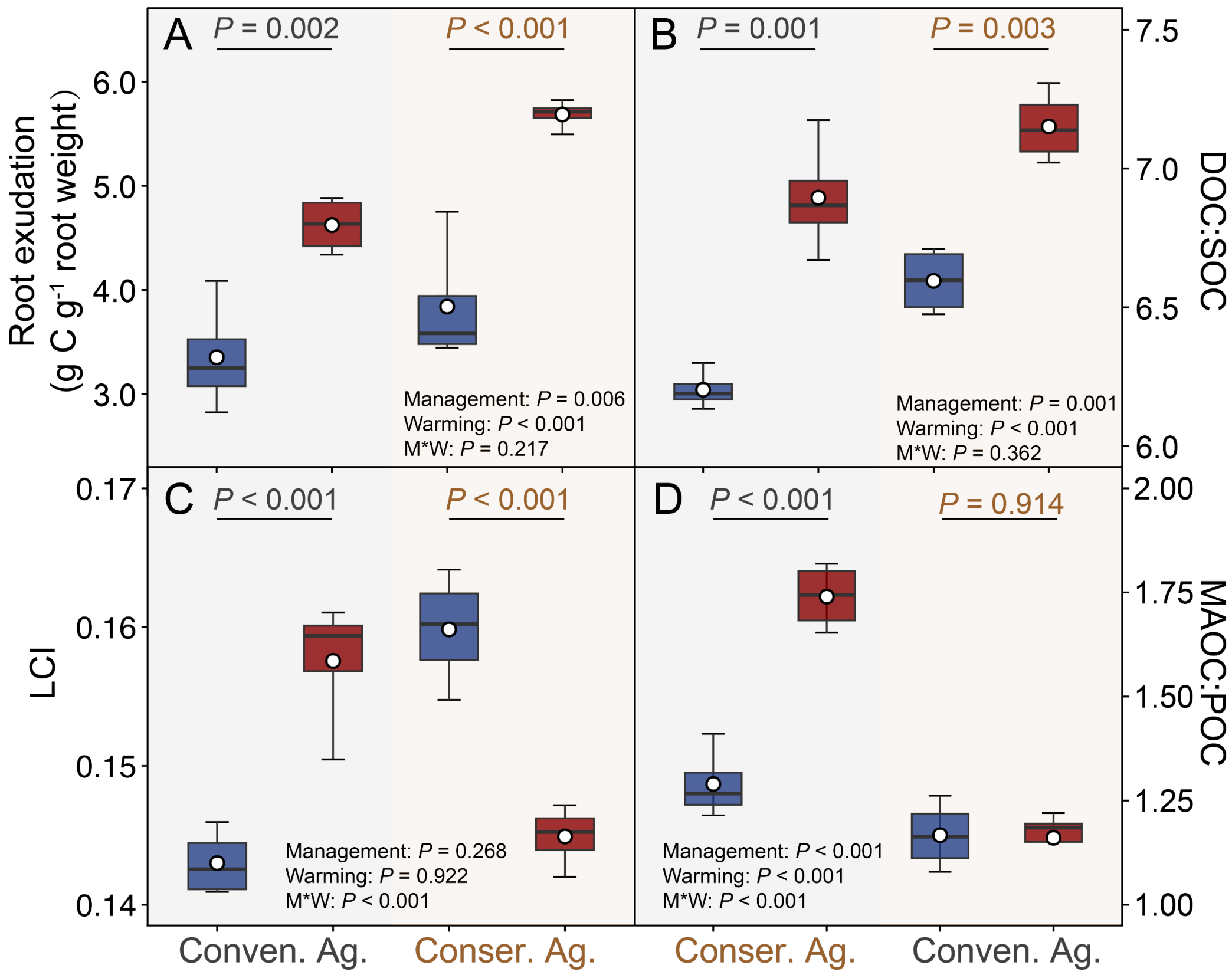

Figure S3. Effects of warming and management on substrate accessibility and quality. Root exudation (A) and DOC:SOC (B) represent substrate accessibility proxies. Lignocellulose index, LCI (C) and MAOC:POC (D) represent substrate quality proxies. The ends of the boxes represent the 25th and 75th percentiles, the horizontal lines inside each box and the whiskers show the median and standard deviations, respectively. The white dot in each box shows the average (n = 4). The effects of management, warming, and their interaction on the response variable were evaluated using linear mixed-effect models with plot as a random effect. Block was included as a random effect to account for experimental design structure. Fixed effects and interaction were evaluated by ANOVA, and post-hoc pairwise comparisons of estimated marginal means were used to examine warming effects within each management system. DOC: dissolved organic carbon; SOC: soil organic carbon; LCI: lignocellulose index; POC: particulate organic carbon; MAOC: mineral-associated organic carbon. Conven. Ag., conventional agriculture; Conser. Ag., conservation agriculture. Data for root exudation carbon input (Oct 2019–May 2020 wheat growth) were from a previously published dataset [9].

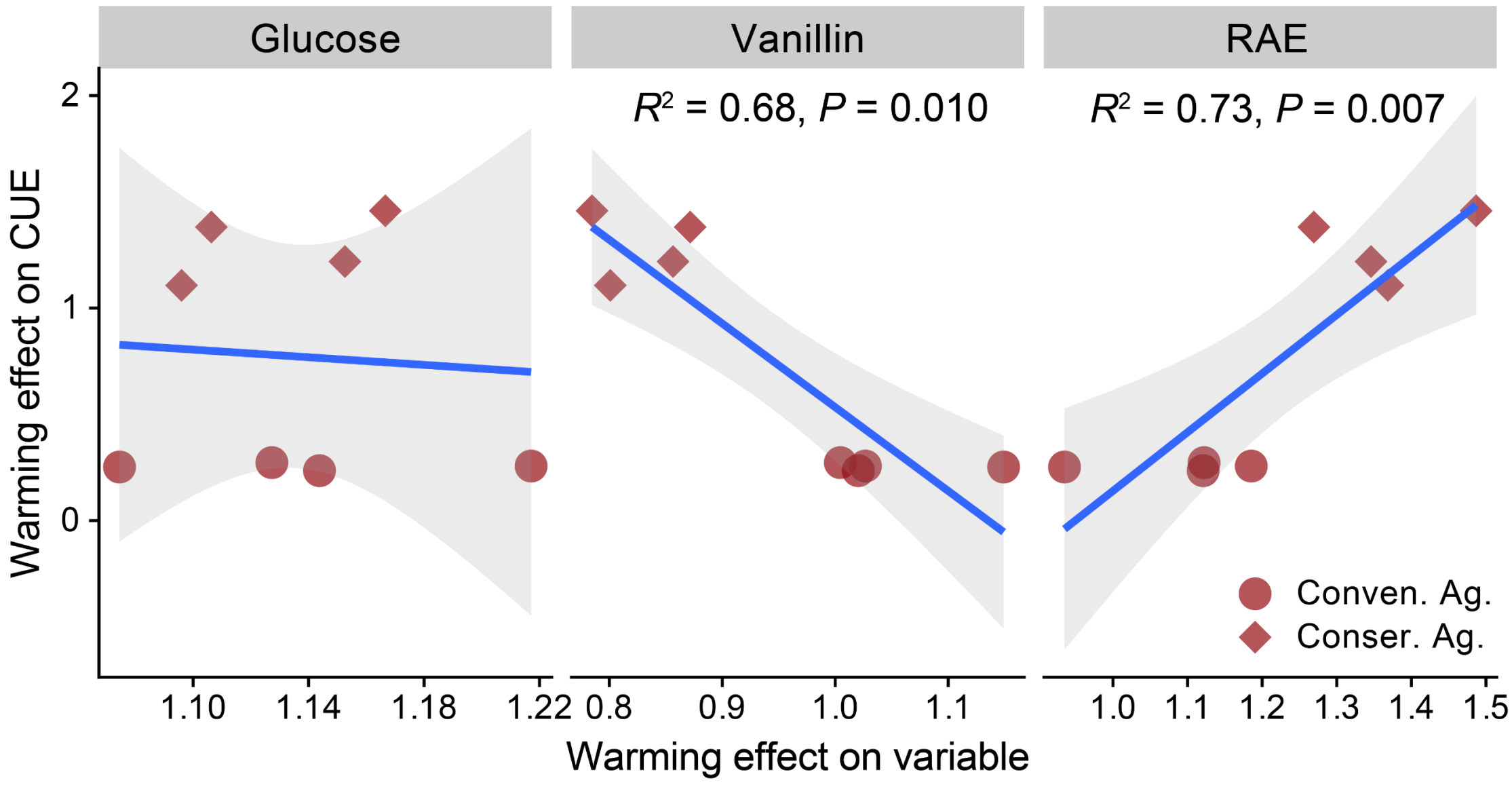

Figure S4. Linking warming-induced shifts in substrate utilization to microbial CUE responses. Linear regressions showing the relationship between the warming effect on community-level CUE and the corresponding warming effects on ^{13}C-glucose utilization efficiency (left), ^{13}C-vanillin utilization efficiency (middle), and relative anabolic efficiency (RAE, right). The warming effect is expressed as the response ratio (Warming/Ambient). Circles and diamonds represent conventional and conservation agricultural, respectively (*n* = 4). Solid blue lines indicate linear regression fits across both management systems, with grey shaded areas representing the 95% confidence intervals; regression lines are only displayed for panels where correlations are statistically significant ($P < 0.05$). CUE, carbon use efficiency; RAE, relative anabolic efficiency; Conven. Ag., conventional agriculture; Conser. Ag., conservation agriculture.

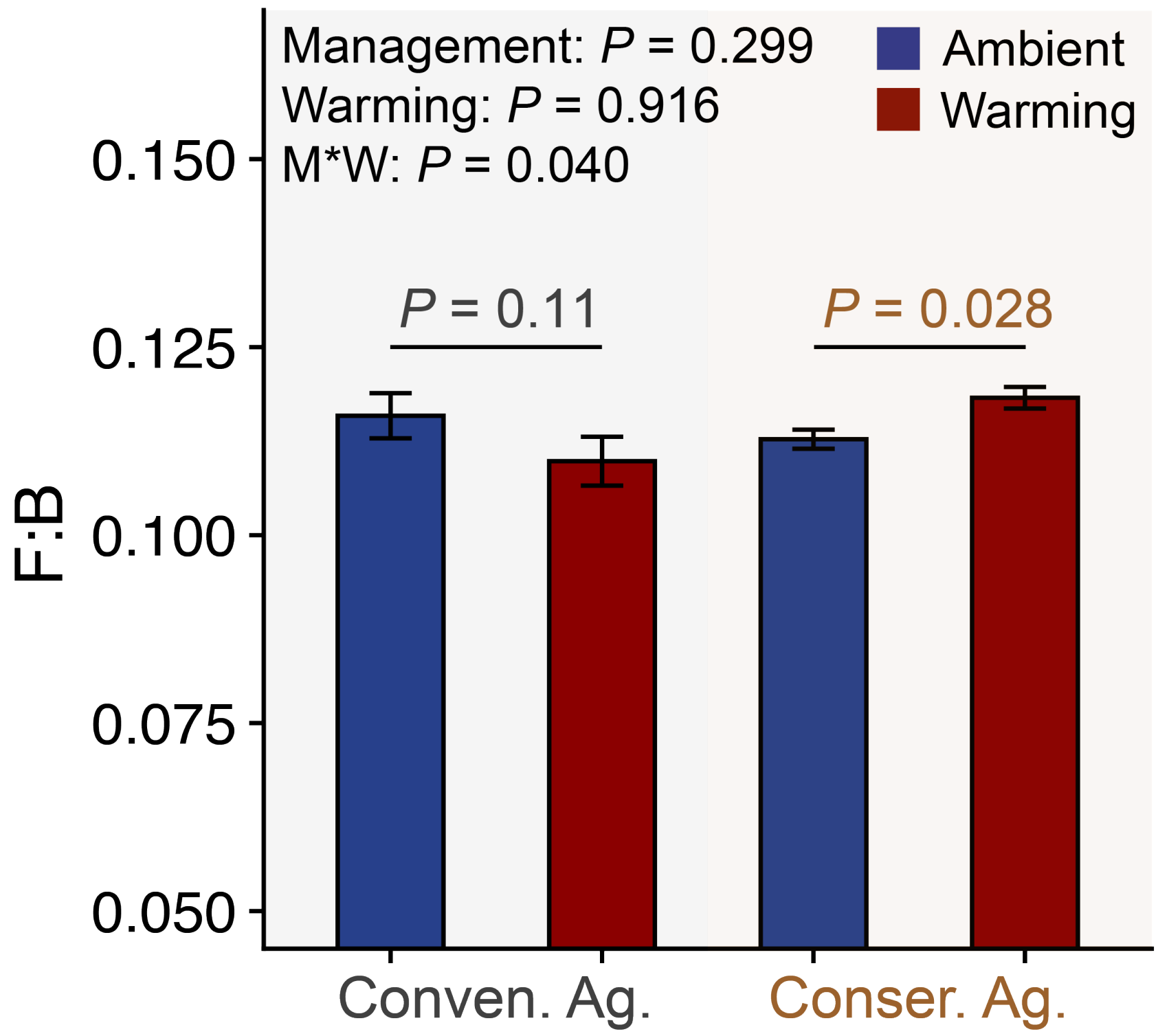

Figure S5. Effects of warming and management on the ratio of fungal to bacterial biomass. Results are presented as mean values ± s.e.m. (*n* = 4). The effects of management, warming, and their interaction on the response variable were evaluated using linear mixed-effect models with plot as a random effect. Block was included as a random effect to account for experimental design structure. Fixed effects and interaction were evaluated by ANOVA, and post-hoc pairwise comparisons of estimated marginal means were used to examine warming effects within each management system. F:B, the ratio of fungal to bacterial PLFA-based biomass. Conven. Ag., conventional agriculture; Conser. Ag., conservation agriculture.

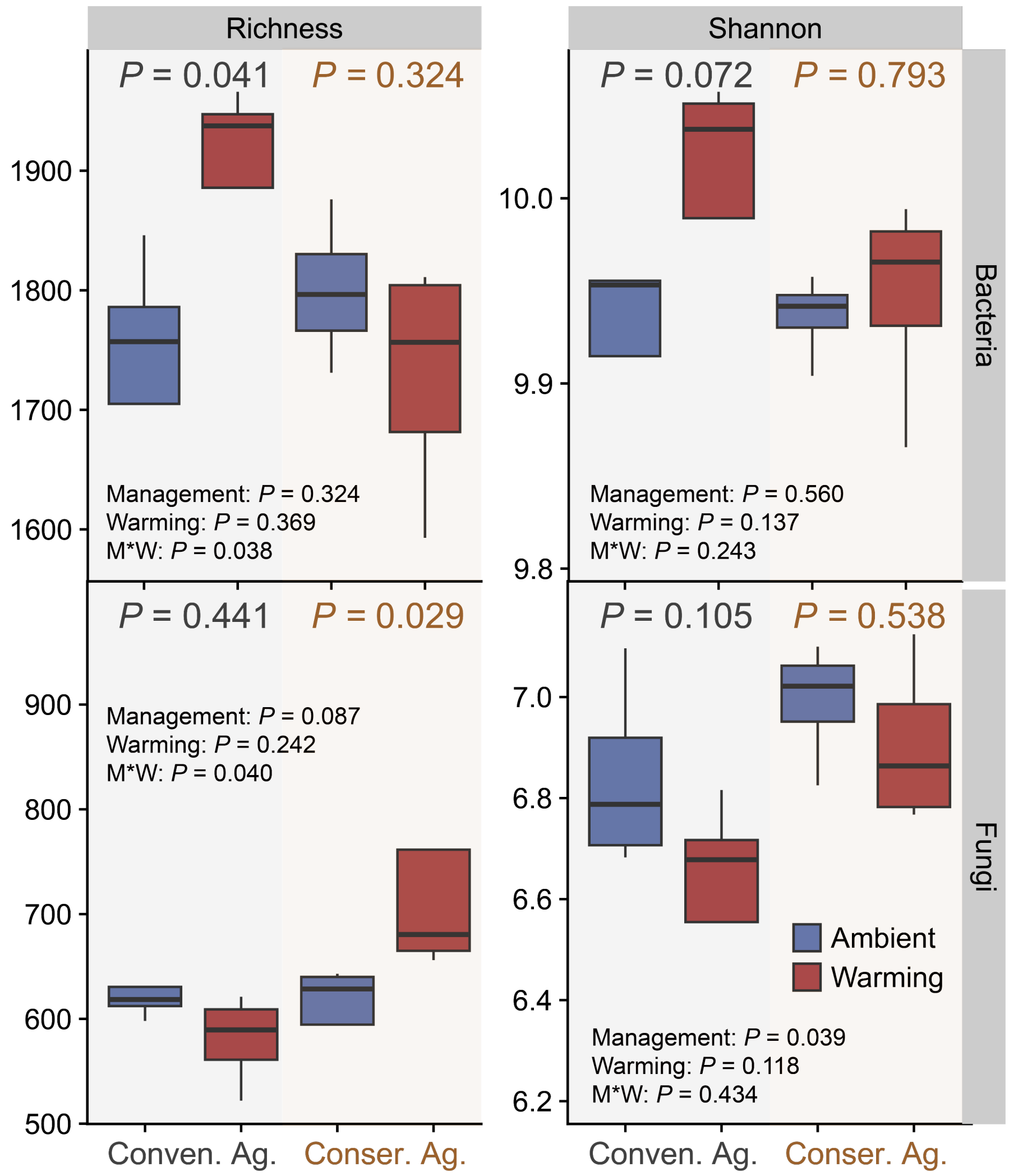

Figure S6. Effects of warming and management on microbial alpha diversity. The ends of the boxes represent the 25th and 75th percentiles, the horizontal lines inside each box and the whiskers show the median and standard deviations, respectively. The effects of management, warming, and their interaction on the response variable were evaluated using linear mixed-effect models with plot as a random effect. Block was included as a random effect to account for experimental design structure. Fixed effects and interaction were evaluated by ANOVA, and post-hoc pairwise comparisons of estimated marginal means were used to examine warming effects within each management system. *n.s.* indicates no significant difference ($P > 0.05$). Richness, observed ASVs; Shannon, Shannon–Wiener index. Conven. Ag., conventional agriculture; Conser. Ag., conservation agriculture.

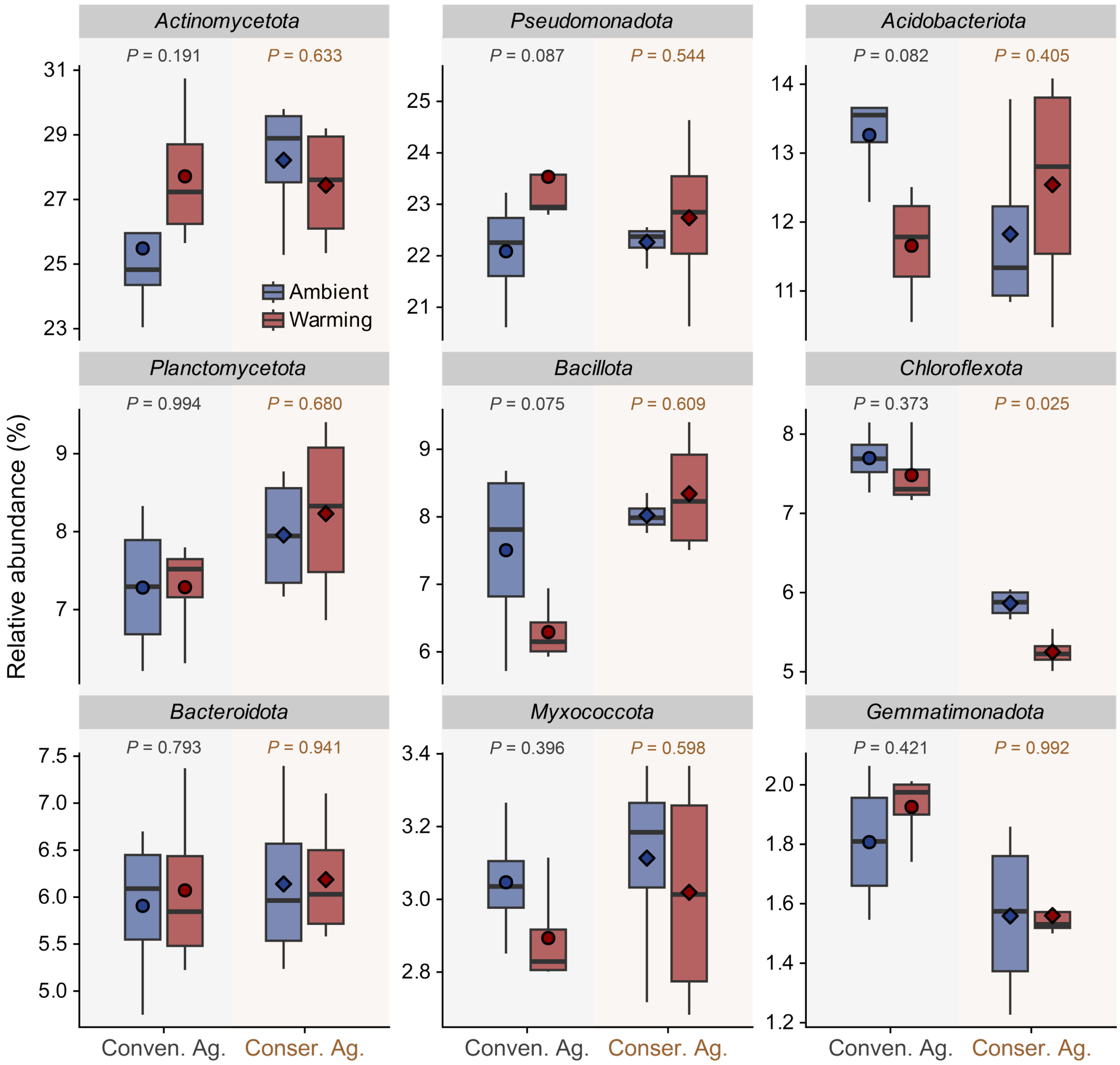

	Management (M)	Warming (W)	M*W
Actinomycetota	*P* = 0.299	*P* = 0.530	*P* = 0.209
Pseudomonadota	*P* = 0.576	*P* = 0.105	*P* = 0.386
Acidobacteriota	*P* = 0.644	*P* = 0.463	*P* = 0.076
Planctomycetota	*P* = 0.109	*P* = 0.766	*P* = 0.744
Bacillota	*P* = 0.014	*P* = 0.323	*P* = 0.106
Chloroflexota	*P* < 0.001	*P* = 0.030	*P* = 0.249
Bacteroidota	*P* = 0.696	*P* = 0.812	*P* = 0.893
Myxococcota	*P* = 0.450	*P* = 0.335	*P* = 0.813
Gemmatimonadota	*P* = 0.012	*P* = 0.561	*P* = 0.570

Figure S7. Effects of warming and management on soil bacterial phylum distribution. The ends of the boxes represent the 25th and 75th percentiles, the horizontal lines inside each box and the whiskers show the median and standard deviations, respectively (n = 4). The effects of management, warming, and their interaction on the response variable were evaluated using linear mixed-effect models with plot as a random effect. Block was included as a random effect to account for

experimental design structure. Fixed effects and interaction were evaluated by ANOVA, and post-hoc pairwise comparisons of estimated marginal means were used to examine warming effects within each management system. The table shown below the figure summarizes the main effects of warming and management, as well as their interaction, on the relative abundances of different bacterial phyla. Conven. Ag., conventional agriculture; Conser. Ag., conservation agriculture.

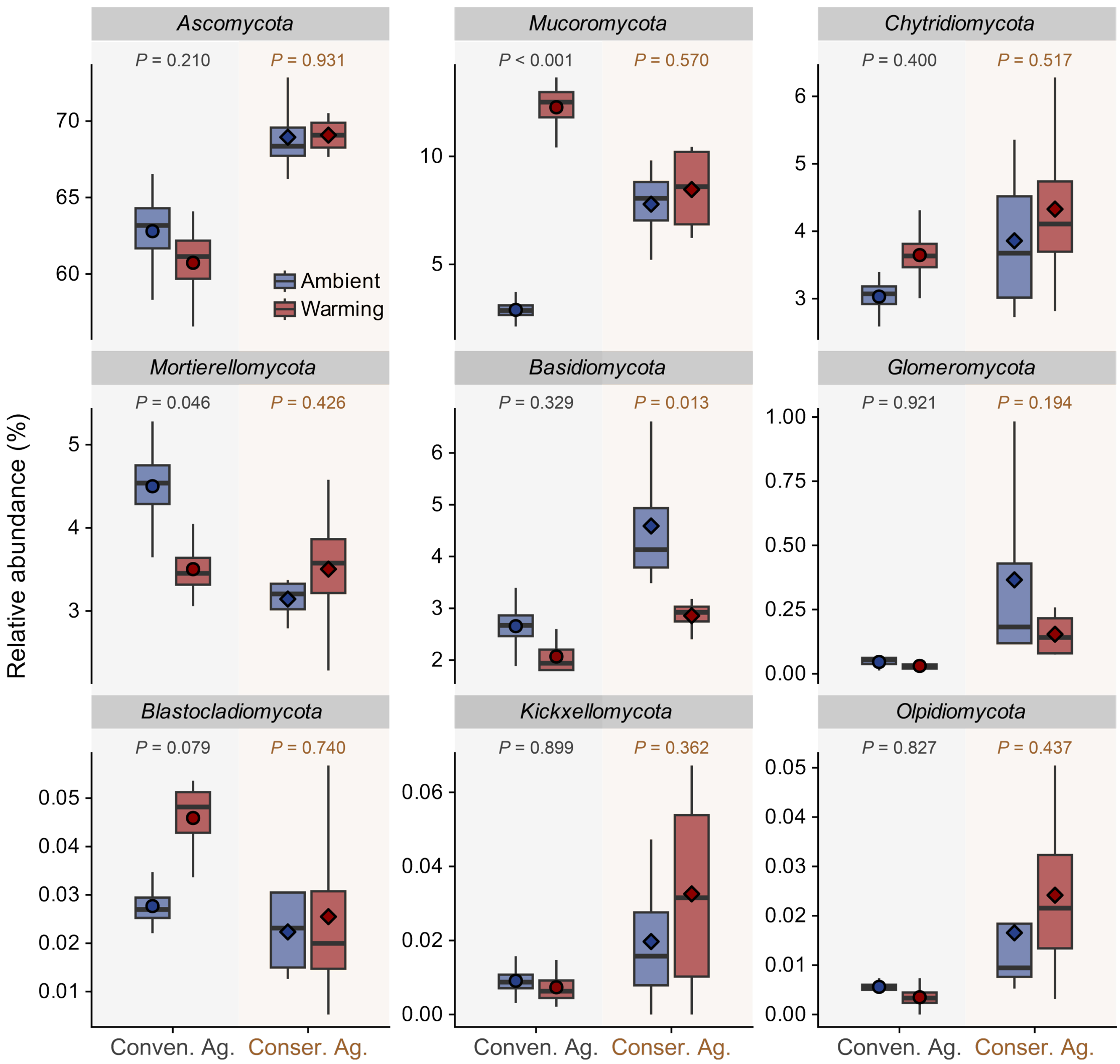

	Management (M)	Warming (W)	M*W
Ascomycota	*P* < 0.001	*P* = 0.395	*P* = 0.335
Mucoromycota	*P* = 0.521	*P* < 0.001	*P* < 0.001
Chytridiomycota	*P* = 0.159	*P* = 0.299	*P* = 0.885
Mortierellomycota	*P* = 0.053	*P* = 0.323	*P* = 0.053
Basidiomycota	*P* = 0.007	*P* = 0.186	*P* = 0.020
Glomeromycota	*P* = 0.067	*P* = 0.314	*P* = 0.381
Blastocladiomycota	*P* = 0.079	*P* = 0.135	*P* = 0.277
Kickxellomycota	*P* = 0.091	*P* = 0.571	*P* = 0.459
Olpidiomycota	*P* = 0.040	*P* = 0.686	*P* = 0.481

Figure S8. Effects of warming and management on soil fungal phylum distribution. The ends of the boxes represent the 25th and 75th percentiles, the horizontal lines inside each box and the whiskers show the median and standard deviations, respectively (n = 4). The effects of management, warming, and their interaction on the response variable were evaluated using linear mixed-effect models with plot as a random effect. Block was included as a random effect to account for

experimental design structure. Fixed effects and interaction were evaluated by ANOVA, and post-hoc pairwise comparisons of estimated marginal means were used to examine warming effects within each management system. The table shown below the figure summarizes the main effects of warming and management, as well as their interaction, on the relative abundances of different fungal phyla. Conven. Ag., conventional agriculture; Conser. Ag., conservation agriculture.

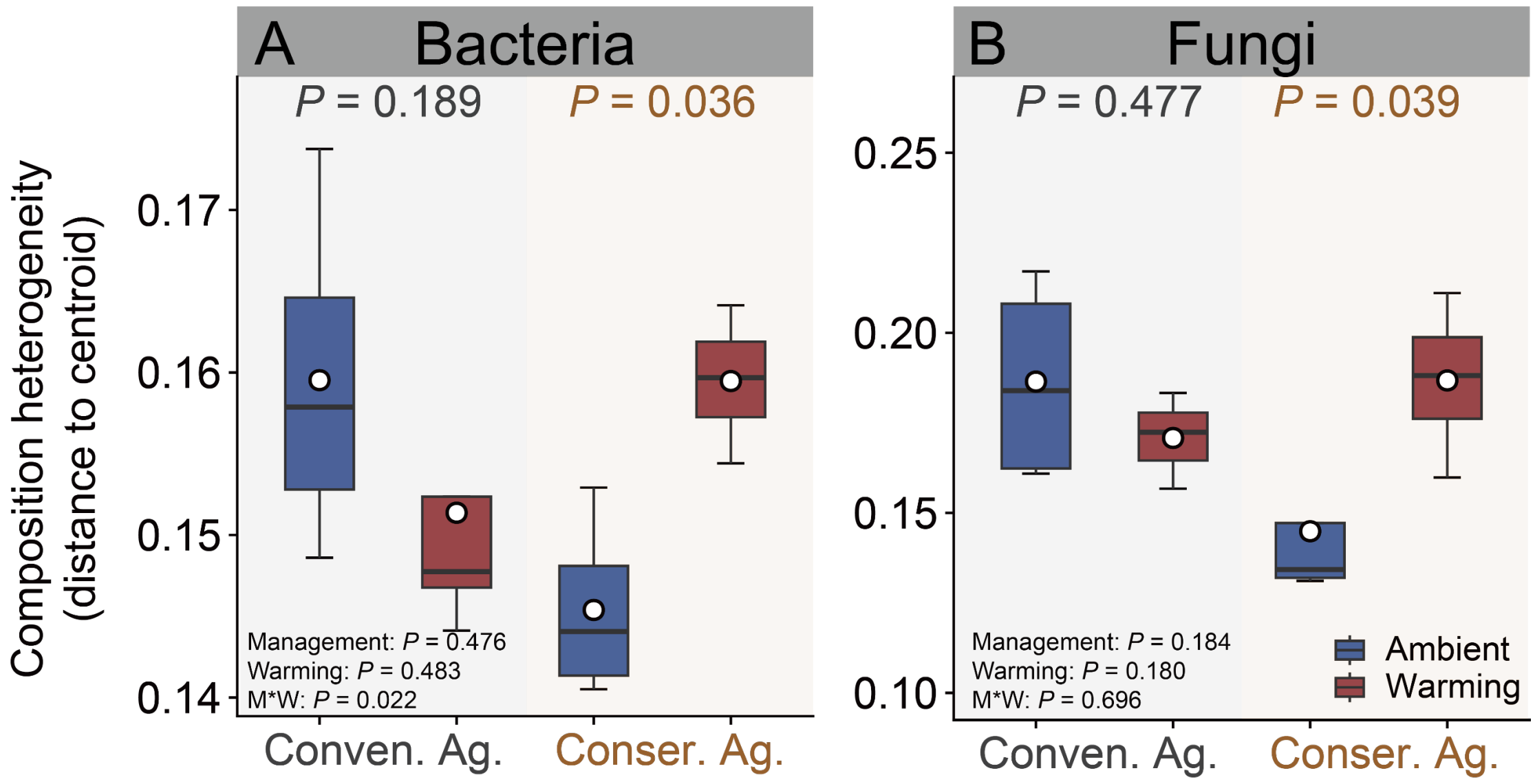

Figure S9. Effects of warming and management on compositional heterogeneity of bacterial (A) and fungal (B) communities. The ends of the boxes represent the 25th and 75th percentiles, and the whiskers refer to the 25th and 75th quartiles (n = 4). The effects of management, warming, and their interaction on the response variable were evaluated using linear mixed-effect models with plot as a random effect. Block was included as a random effect to account for experimental design structure. Fixed effects and interaction were evaluated by ANOVA, and post-hoc pairwise comparisons of estimated marginal means were used to examine warming effects within each management system. Only significant differences between treatments are shown. Conven. Ag., conventional agriculture; Conser. Ag., conservation agriculture.

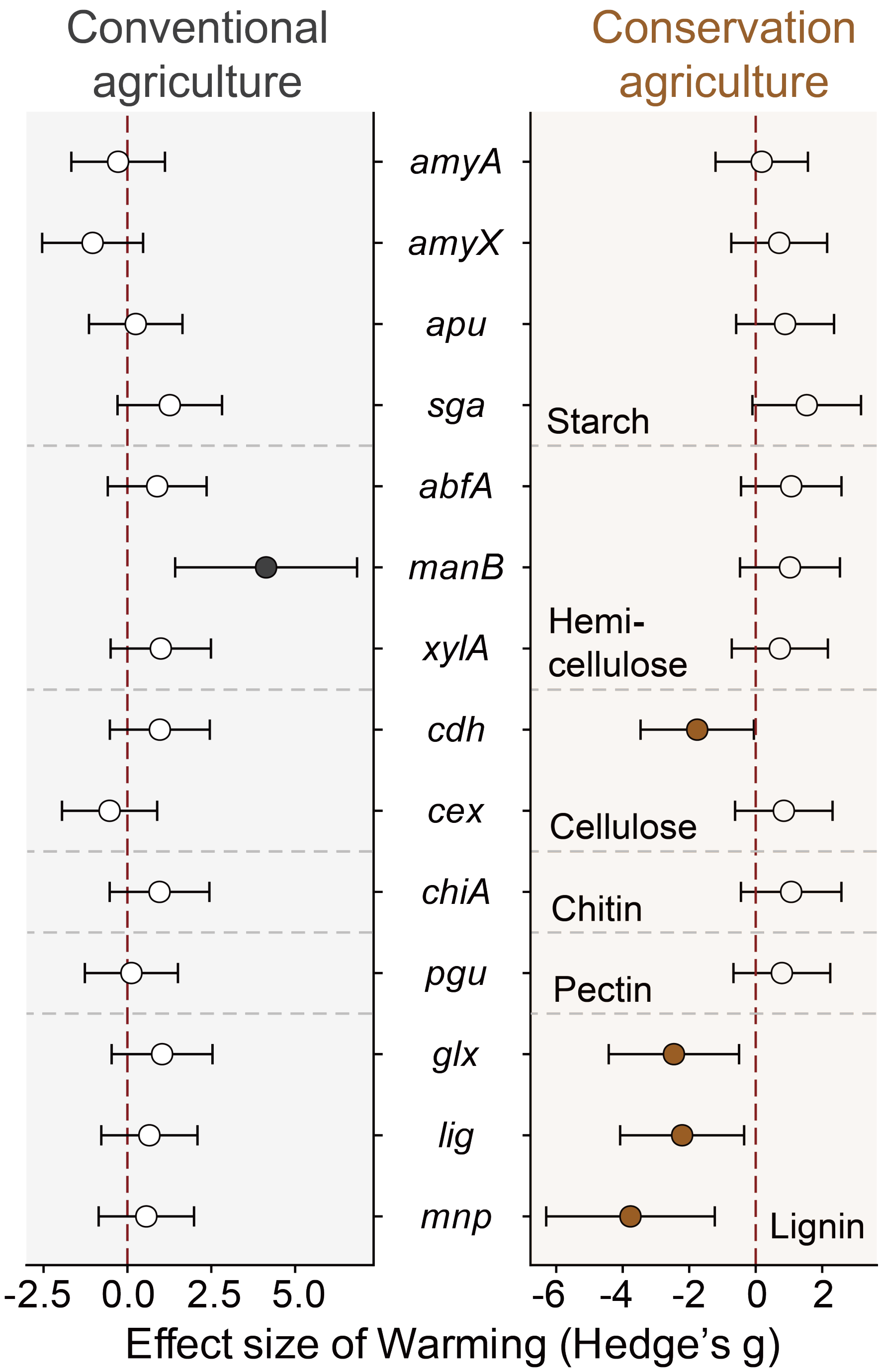

Figure S10. Effects of warming on the absolute abundance of carbon degradation genes (via QMEC) in conventional and conservation agriculture. Changes in C degradation gene abundances triggered by warming as expressed by Hedges' g. Solid circles indicate significant differences between warming and ambient conditions (*P* < 0.05), while open circles represent non-significant differences. The complexity of C is presented in order from labile to stable C.

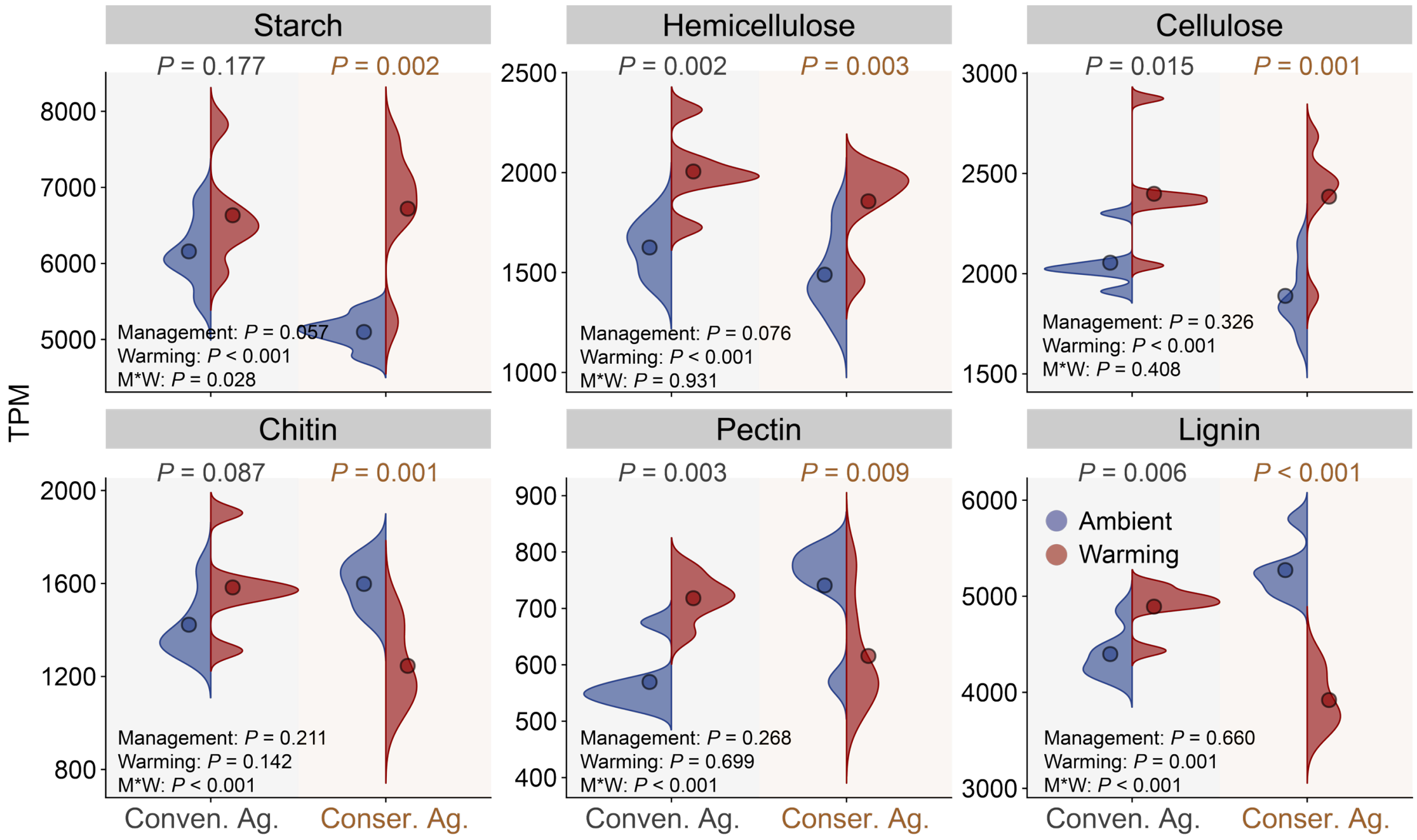

Figure S11. Effects of warming and management on microbial functional genes for polymeric C degradation based on the Kyoto Encyclopedia of Genes and Genomes (KEGG) database. Data are presented as violin plots with mean values ± s.e.m. (n = 4). Circles within violins show mean values. The effects of management, warming, and their interaction on the response variable were evaluated using linear mixed-effect models with plot as a random effect. Block was included as a random effect to account for experimental design structure. Fixed effects and interaction were evaluated by ANOVA, and post-hoc pairwise comparisons of estimated marginal means were used to examine warming effects within each management system. Conven. Ag., conventional agriculture; Conser. Ag., conservation agriculture.

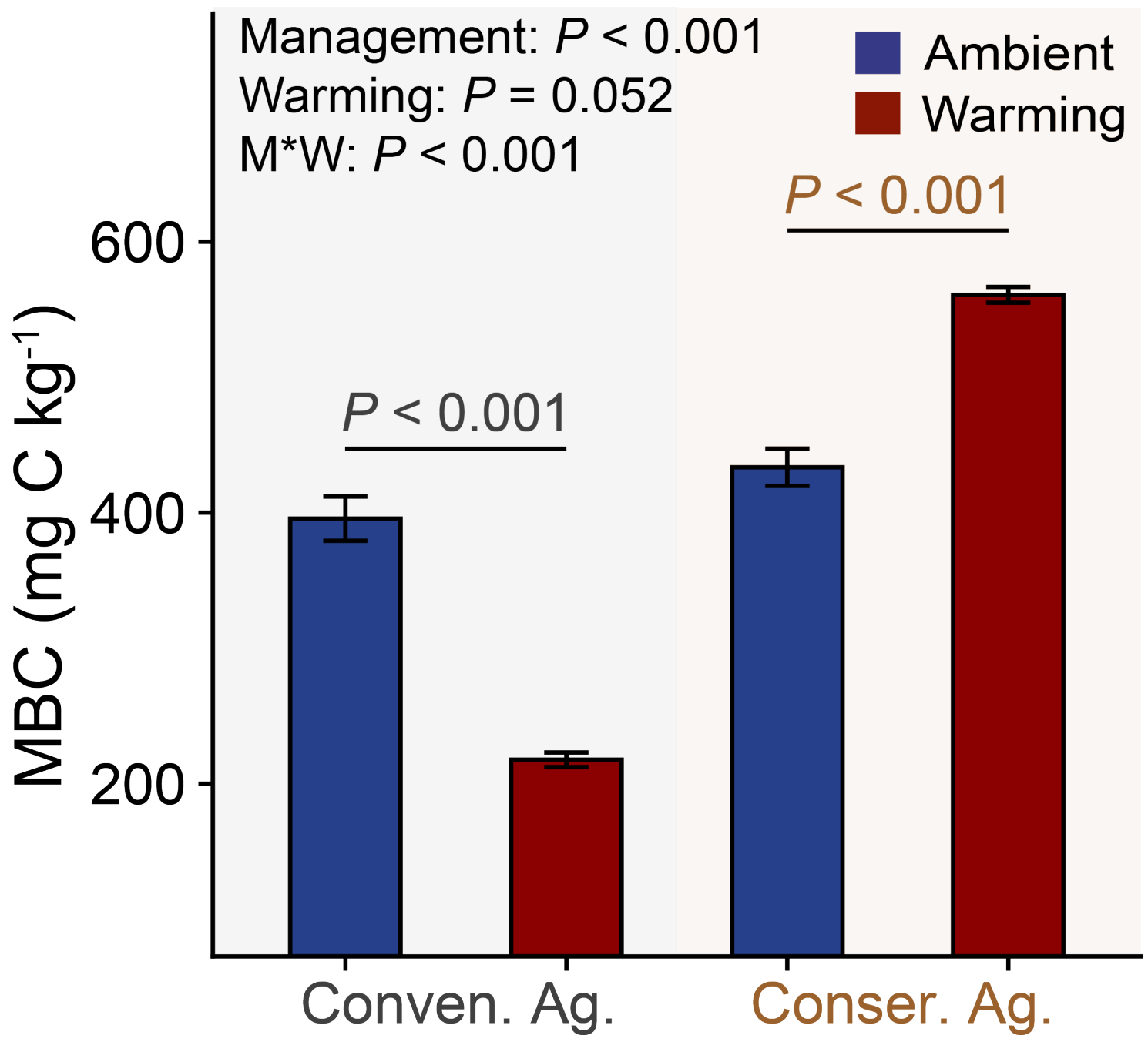

Figure S12. Effects of warming and management on microbial biomass carbon (MBC). Results are presented as mean values ± s.e.m. (*n* = 4). The effects of management, warming, and their interaction on the response variable were evaluated using linear mixed-effect models with plot as a random effect. Block was included as a random effect to account for experimental design structure. Fixed effects and interaction were evaluated by ANOVA, and post-hoc pairwise comparisons of estimated marginal means were used to examine warming effects within each management system. Conven. Ag., conventional agriculture; Conser. Ag., conservation agriculture.

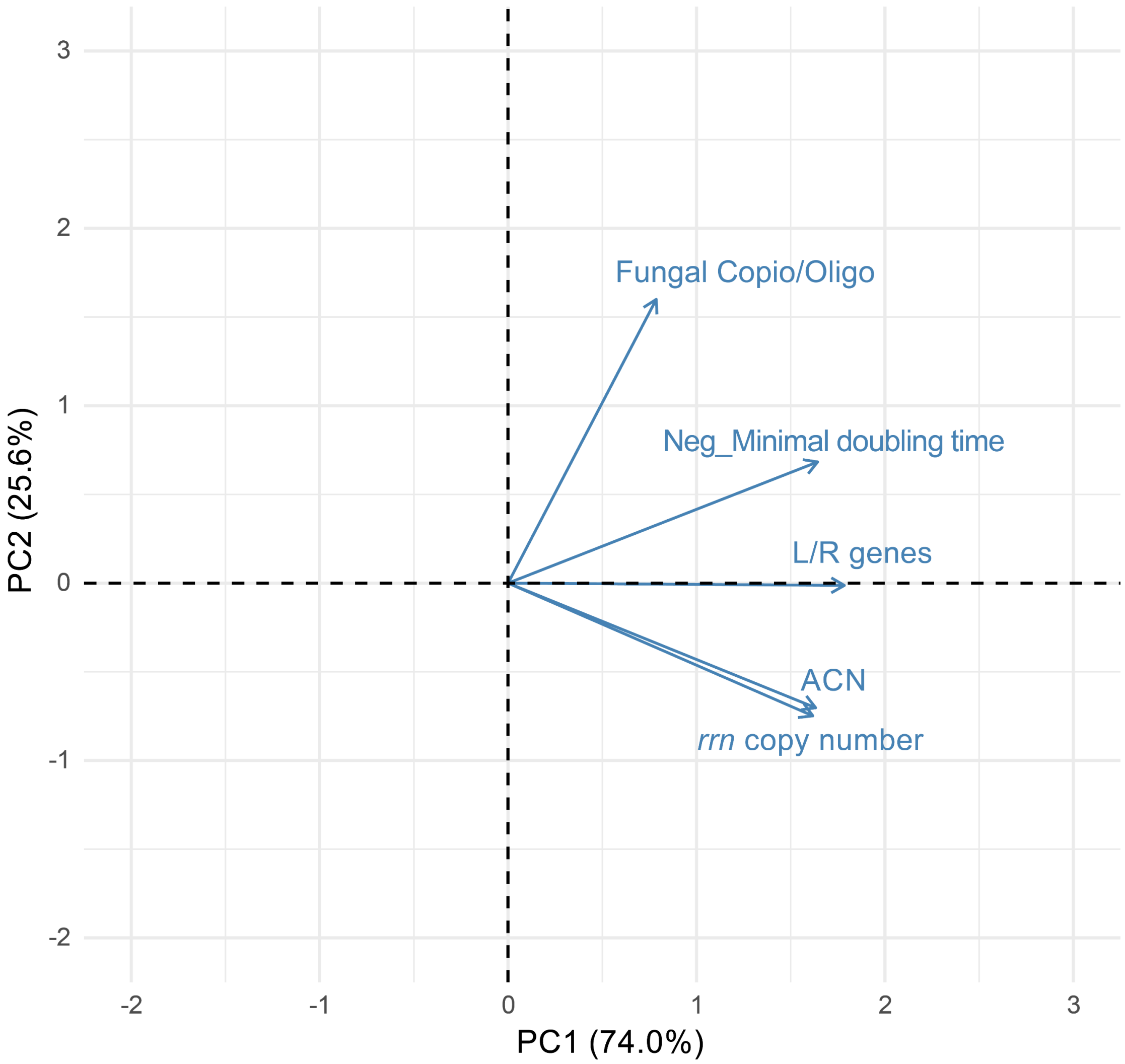

Figure S13. Microbial life-history strategies. PCA loading plot of microbial life-history strategies based on principal component analysis of five trait indicators: the fungal copiotrophic/oligotrophic ratio (copio/oligo), the community-weighted mean bacterial rRNA operon copy number (rrn copy number), the ratio of labile to recalcitrant carbon degradation gene abundance (L/R genes), the reciprocal of minimal doubling time and average 16S rRNA copy number (ACN).

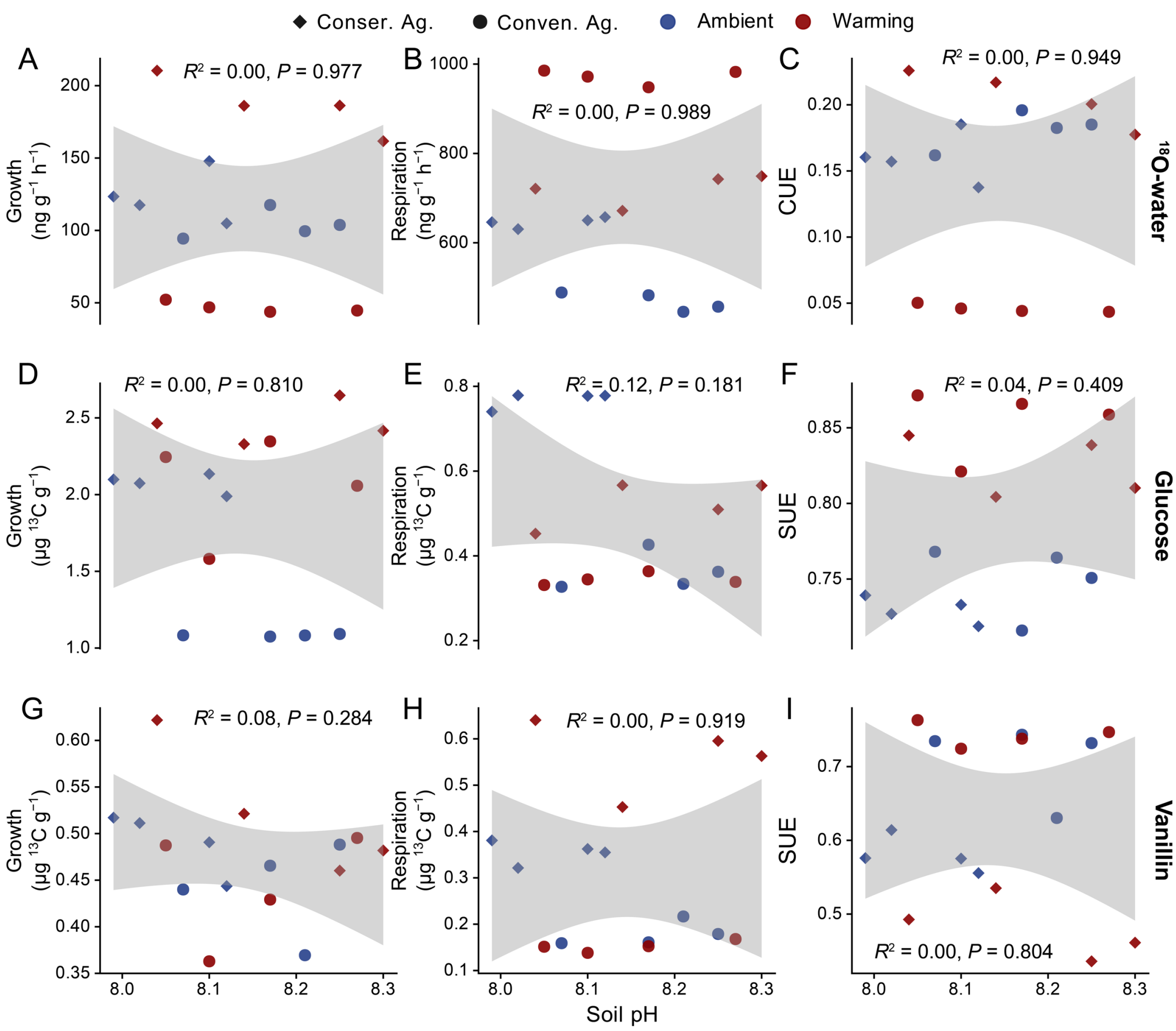

Figure S14. Relationships between soil pH and microbial carbon allocation and carbon use efficiency under warming and agricultural management. Panels show the relationships between soil pH and microbial growth, respiration, carbon use efficiency (CUE), and substrate-specific use efficiency (SUE) based on ^{18}O-water and ^{13}C-substrate tracing. Panels A–C show ^{18}O-water-derived microbial growth, respiration and CUE. Panels D–I show glucose- and vanillin-derived microbial growth, respiration and SUE, respectively. Shapes indicate agricultural management, with circles representing conventional agriculture and diamonds representing conservation agriculture. Colors indicate temperature treatment, with blue representing ambient temperature and red representing warming. Grey bands indicate linear regression fits and 95% confidence intervals. R^2 and P values are shown for each relationship.

Table S1. Chemical, thermodynamic and metabolic indicators supporting glucose and vanillin as contrasting substrates in the substrate-specific carbon use efficiency assay.

Indicators	Glucose	Vanillin	Theoretical implication for apparent bioavailability	Reference
Chemical class	non-aromatic monosaccharide	lignin-derived aromatic aldehyde	Indirect; monosaccharides are generally more readily metabolized, whereas aromatic compounds usually require specialized catabolic pathways	
Molecular formula	$C_6H_{12}O_6$	$C_8H_8O_3$	Descriptive; no direct directional implication	
Molar mass	180.16 g mol^{-1}	152.15 g mol^{-1}	Descriptive; molecular size alone does not determine bioavailability	
Carbon content	40.0%	63.1%	Descriptive; higher C content does not necessarily indicate higher bioavailability	
Aromatic structure	No	Yes	−; aromaticity generally increases biochemical complexity and the need for specialized degradation	

Solubility	high	moderate	+ for glucose / − for vanillin; higher solubility generally increases substrate mobility and microbial access	
XLogP3 / LogP	−2.6	1.2	− for vanillin; higher LogP indicates greater hydrophobicity and potentially lower aqueous availability	[10–12]
NOSC	0.00	−0.25	Context-dependent; lower NOSC may provide more thermodynamic energy upon oxidation, but does not necessarily increase apparent bioavailability	[13, 14]
Relative hydrophobicity / sorption potential	Low	Higher than glucose	− for vanillin; higher hydrophobicity and sorption potential may reduce mobility and microbial access	[15]
Entry into central metabolism	Direct entry via glycolysis and related central carbon pathways	Requires peripheral aromatic-catabolic conversion before entry into central metabolism	+ for glucose / − for vanillin; direct entry into central metabolism indicates higher biochemical accessibility	[16, 17]

Main metabolic interpretation	Readily available carbohydrate providing energy, reducing power and biosynthetic precursors	Lignin-derived aromatic substrate requiring specialized degradation and funneling pathways	Integrative interpretation; glucose is expected to have higher apparent bioavailability than vanillin	[18, 19]

Glucose was used as a readily available non-aromatic monosaccharide, whereas vanillin was used as a lignin-derived aromatic aldehyde that differs from glucose in aromaticity, hydrophobicity, sorption potential, carbon oxidation state and metabolic accessibility. XLogP3 / LogP values were obtained from PubChem computed properties and were used as chemical proxies for hydrophobicity and potential sorption to organic surfaces, rather than as sole determinants of substrate bioavailability. Nominal oxidation state of carbon (NOSC) was calculated from molecular formulae as an indicator of the redox state and thermodynamic properties of substrate C. The lower NOSC of vanillin indicates that it is more reduced than glucose; however, microbial growth on vanillin also requires specialized aromatic-catabolic reactions before its C can enter central metabolism. “+” and “−” indicate the expected positive or negative effects on apparent bioavailability, respectively. “Context-dependent” indicates that the direction cannot be inferred from this property alone. Together, these indicators support the interpretation of glucose and vanillin as chemically, thermodynamically and metabolically contrasting substrates, rather than as a strict labile-versus-recalcitrant dichotomy.

Table S2. Copiotrophic- and oligotrophic-associated bacterial/fungal phyla defined in the study.

	Phylum (Subphylum)	Trophic lifestyle	References
Bacteria	*α- Pseudomonadota*	Oligotroph	[20]
	γ- Pseudomonadota	Copiotroph	[21]
	Acidobacteriota	Oligotroph	[22]
	Actinobacteriota	Copiotroph	[20]
	Bacteroidota	Copiotroph	[23]
	Chloroflexota	Oligotroph	[24]
	Bacillota	Copiotroph	[25]
	Planctomycetota	Oligotroph	[23]
	Verrucomicrobiota	Oligotroph	[26]
Fungi	*Ascomycota*	Copiotroph	[23, 27]
	Basidiomycota	Oligotroph	

To ensure taxonomic consistency, the microbial nomenclature used in this study adheres to the latest phylogenetic classifications. Specifically, we have updated several phylum-level identifiers to align with recent taxonomic standards: *Pseudomonadota*, *Bacillota*, and *Planctomycetota* have been adopted to replace the traditional names *Proteobacteria*, *Firmicutes*, and *Planctomycetes*, respectively. These revisions follow the standard naming convention requiring the suffix '-ota' for phylum-level designations, with many names now rooted in their respective type genera (e.g., *Planctomyces*).

Table S3. Main and interactive effects of management systems and warming on microbial carbon metabolism, substrate traits, microbial traits and edaphic conditions.

Variables	Management	Warming	Management × Warming
CUE (^{18}O-H_2O)	**72.7 (< 0.001)**	**31.1 (< 0.001)**	**124 (< 0.001)**
Respiration (^{18}O-H_2O)	**12.3 (0.006)**	**770 (< 0.001)**	**422 (< 0.001)**
Growth (^{18}O-H_2O)	**125 (< 0.001)**	0.16 (0.693)	**71.0 (< 0.001)**
CUE (^{13}C-Glu)	**6.40 (0.032)**	**101 (< 0.001)**	0.234 (0.640)
Respiration (^{13}C-Glu)	**245 (< 0.001)**	**49.5 (< 0.001)**	**36.9 (< 0.001)**
Growth (^{13}C-Glu)	**57.0 (< 0.001)**	**54.3 (< 0.001)**	**9.97 (0.011)**
CUE (^{13}C-Van)	**110 (< 0.001)**	3.14 (0.101)	**12.5 (0.004)**
Respiration (^{13}C-Van)	**230 (< 0.001)**	**22.1 (0.001)**	**36.8 (< 0.001)**
Growth (Van)	**5.13 (0.049)**	0.35 (0.565)	0.24 (0.633)
RAE	**55.8 (< 0.001)**	**28.7 (< 0.001)**	**13.3 (0.005)**
Root exudation	**12.7 (0.006)**	**51.4 (< 0.001)**	1.75 (0.217)
DOC:SOC	**21.1 (0.001)**	**78.3 (< 0.001)**	0.92 (0.362)
LCI	1.38 (0.268)	0.01 (0.922)	**68.9 (< 0.001)**
MAOC:POC	**76.5 (< 0.001)**	**30.5 (< 0.001)**	**32.3 (< 0.001)**
Community-level *rrn* copy numbers	5.05 (0.051)	4.06 (0.074)	**6.58 (0.030)**
Bacterial Copio/Oligo	**8.43 (0.017)**	1.21 (0.299)	**9.90 (0.011)**
Fungal Copio/Oligo	**24.8 (< 0.001)**	**5.37 (0.045)**	0.19 (0.671)
L/R genes	1.69 (0.224)	**20.4 (0.001)**	**13.9 (0.004)**
Anabolism/Catabolism	**11.7 (0.007)**	**5.86 (0.038)**	**12.0 (0.007)**
AGS	2.21 (0.181)	0.16 (0.698)	5.52 (0.051)
ACN	**12.0 (0.010)**	**11.2 (0.012)**	**6.50 (0.038)**
CUBHE	2.52 (0.155)	0.29 (0.602)	0.30 (0.599)
Minimal doubling time	0.29 (0.601)	4.79 (0.064)	3.05 (0.124)
Soil moisture	**64.9 (< 0.001)**	**177 (< 0.001)**	2.85 (0.125)
Soil temperature	**84.4 (< 0.001)**	**543 (< 0.001)**	**6.70 (0.029)**
F:B	1.21 (0.299)	0.012 (0.916)	**5.72 (0.040)**

MBC	**280 (< 0.001)**	**4.96 (0.052)**	**179 (< 0.001)**

Results of mixed-effects models testing management, warming, and their interaction on soil variables. The results are presented as *F* values (*P* values). Significant effects (*P* < 0.05) are highlighted in bold. Glu, glucose; Van, vanillin. RAE, relative anabolic efficiency; DOC: dissolved organic carbon; SOC: soil organic carbon; LCI: lignocellulose index; POC: particulate organic carbon; MAOC: mineral-associated organic carbon; Bacterial Copio/Oligo, bacterial copiotrophic/oligotrophic taxa ratios; Fungal Copio/Oligo, fungal copiotrophic/oligotrophic taxa ratios; Anabolism/Catabolis, the anabolic/catabolic gene abundance ratio; L/r genes, the labile/recalcitrant C degradation gene abundance ratio; F:B, the ratio of fungal to bacterial PLFA-based biomass; MBC, microbial biomass carbon.

Table S4. Soil physicochemical properties under different treatments.

Edaphic conditions	Conven. Ag. + Ambient	Conven. Ag. + Warming	Conser. Ag. + Ambient	Conser. Ag. + Warming	Management	Warming	Management × Warming
pH	8.18 ± 0.04a	8.15 ± 0.05a	8.06 ± 0.03a	8.18 ± 0.06a	0.338	0.263	0.094
SOC	8.53 ± 0.10c	8.43 ± 0.16c	13.0 ± 0.18b	13.6 ± 0.21a	**< 0.001**	**< 0.001**	0.094
TN	0.97 ± 0.03c	1.03 ± 0.01c	1.48 ± 0.04b	1.62 ± 0.04a	**< 0.001**	**0.010**	0.232
DOC	52.89 ± 0.63d	58.10 ± 0.29c	86.85 ± 1.07b	97.81 ± 0.97a	**< 0.001**	**< 0.001**	**< 0.001**
NO_3^--N	12.57 ± 0.26c	16.62 ± 0.37b	17.24 ± 0.51b	22.84 ± 0.57a	**< 0.001**	**< 0.001**	0.100
NH_4^+-N	1.22 ± 0.05a	1.44 ± 0.18a	1.04 ± 0.09a	1.12 ± 0.04a	**0.024**	0.136	0.481

Values are means ± standard errors. Different lowercase letters indicate significant differences among treatments at $P < 0.05$ based on linear mixed-effects models with block as a random effect. The last three columns show P values from two-way linear mixed-effects models with management, warming, and their interaction as fixed effects and block as a random effect. SOC, soil organic carbon; TN, total nitrogen; DOC, dissolved organic carbon. Conven. Ag., conventional agriculture; Conser. Ag., conservation agriculture.

Table S5. Effects of warming and management on bacterial and fungal β-diversity by PERMANOVA, ANOSIM and MRPP

			Management	Warming	Management × Warming
Bacteria	PERMANOVA	R^2	**0.320**	0.060	**0.090**
		P	**0.001**	0.131	**0.033**
	ANOSIM	R	**1.000**	0.020	/
		P	**0.001**	0.241	/
	MRPP	expected delta	**0.310**	0.310	/
		P	**0.001**	0.196	/
Fungi	PERMANOVA	R^2	**0.330**	**0.160**	**0.130**
		P	**0.001**	**0.002**	**0.002**
	ANOSIM	R	**0.800**	**0.290**	/
		P	**0.001**	**0.012**	/
	MRPP	expected delta	**0.440**	**0.440**	/
		P	**0.002**	**0.009**	/

Values represent the estimation of the variance component (R^2) for PERMANOVA and the global test statistic (R) for ANOSIM, as well as expected delta for MRPP. The permutation-based level of significance (*P*) is provided for all tests. Values at $P < 0.05$ are shown in bold. ANOSIM and MRPP do not calculate interaction effects. PERMANOVA, permutational multivariate analysis of variance; ANOSIM, analysis of similarities; MRPP, Multi Response Permutation Procedure.

330 **Table S6. Summary of direct, indirect, and total effects for each response**
331 **variable.**

Response variables	Variables	Total effect (95% CI)	Direct effect (95% CI)	Indirect effect (95% CI)
CUE	Temperature (W)	-0.230 [-0.484, 0.077]	-0.102 [-0.405, 0.180]	-0.128 [-0.345, 0.136]
	Management (M)	-0.014 [-0.244, 0.220]	/	-0.014 [-0.244, 0.220]
	W × M	0.175 [0.085, 0.337]*	/	0.175 [0.085, 0.337]*
	Substrate availability	-0.149 [-0.395, 0.287]	-0.361 [-0.634, -0.035]*	0.212 [0.116, 0.572]*
	Substrate quality	0.261 [0.117, 0.507]*	/	0.261 [0.117, 0.507]*
	Microbial life-history strategies	0.109 [0.009, 0.260] *	/	0.109 [0.009, 0.260] *
	Microbial metabolism	0.332 [0.119, 0.715]*	0.184 [-0.075, 0.680]	0.148 [0.040, 0.349]*
	RAE	0.367 [0.027, 0.653]*	0.367 [0.027, 0.653]*	/
RAE	Temperature (W)	0.223 [0.035, 0.479]*	/	0.223 [0.035, 0.479]*
	Management (M)	0.294 [0.098, 0.548]*	/	0.294 [0.098, 0.548]*
	W × M	0.318 [0.223, 0.543]*	/	0.318 [0.223, 0.543]*
	Substrate availability	0.352 [0.189, 0.625]*	/	0.352 [0.189, 0.625]*
	Substrate quality	0.473 [0.296, 0.758]*	0.089 [-0.127, 0.345]	0.383 [0.221, 0.682]*
	Microbial life-history strategies	0.297 [0.074, 0.573]*	0.297 [0.074, 0.573]*	/
	Microbial metabolism	0.403 [0.148, 0.643]*	0.403 [0.148, 0.643]*	/
Microbial metabolism	Temperature (W)	0.291 [0.000, 0.530]	/	0.291 [0.000, 0.530]
	Management (M)	0.417 [0.132 , 0.674]*	/	0.417 [0.132 , 0.674]*
	W × M	0.318 [0.086, 0.565]*	/	0.318 [0.086, 0.565]*
	Substrate availability	0.446 [0.029, 0.707]*	0.446 [0.029, 0.707]*	/
	Substrate quality	0.474 [0.123, 0.704]*	0.474 [0.123, 0.704]*	/
Microbial life-history strategies	Temperature (W)	0.377 [0.046, 0.668]*	/	0.377 [0.046, 0.668]*
	Management (M)	0.318 [-0.058, 0.585]	-0.237 [-0.548, 0.077]	0.554 [0.271, 0.851]*
	W × M	0.436 [0.223, 0.701]*	/	0.436 [0.223, 0.701]*
	Substrate availability	0.582 [0.348, 0.817]*	0.582 [0.348, 0.817]*	/
	Substrate quality	0.649 [0.391, 0.867]*	0.649 [0.391, 0.867]*	/
Substrate availability	Temperature (W)	0.730 [0.466, 0.875]*	0.730 [0.466, 0.875]*	/
	Management (M)	0.552 [0.243, 0.770]*	0.552 [0.243, 0.770]*	/

Substrate quality	Temperature (W)	-0.073 [-0.515, 0.381]	-0.073 [-0.515, 0.381]	/
	Management (M)	0.360 [-0.117, 0.650]	0.360 [-0.117, 0.650]	/
	W × M	0.672 [0.273, 0.882]*	0.672 [0.273, 0.882]*	/

Asterisks (*) denote significant effects where the confidence intervals exclude zero. CIs were derived from 9999 bootstrap iterations (R = 9999) using the semEff package, with a fixed seed (23) for reproducibility.

Table S7. List of the CAZy families involved in microbial functional potentials.

CAZy gene family	Functions	References
GT39	Cell membrane/wall components	[28]
GT26	Cell membrane/wall components	[28]
GH31	Starch decomposition	[28]
GT51	murein synthesis	[29]
GT28	galactolipid biosynthesis	[29]
GT20	trehalose synthesis, osmolyte synthesis	[29]
GH5	Cellulose decomposition	[29]
GH29	Hemicellulose decomposition	[30]
GH32	Starch decomposition	[31]
GH20	Chitin decomposition	[32]
GH43	Hemicellulose/Pectin decomposition	[33]
GH13	Starch decomposition	[31]
GT4	Cell membrane/wall components	[28]
GH3	Cellulose decomposition	[33]
GH51	Hemicellulose decomposition	[29]
GT2	Cell membrane/wall components	[28]
GT35	Starch decomposition	[29]
GH54	Hemicellulose decomposition	[34]
GH15	Starch decomposition	[31]
CBM48	Cellulose/hemicellulose-binding function	[35]
CBM42	Lignocellulose-binding function	[36]
GH9	Hemi-/Cellulose decomposition	[32]

Table S8. Classification of ECs selected for anabolism and catabolism.

Metabolism process	Pathway	Code of enzymes
Catabolism	EMP	5.1.3.3, 2.7.1.1, 2.7.1.2, 2.7.1.63, 2.7.1.147, 5.1.3.15, 5.3.1.9, 5.4.2.2, 2.7.1.90, 2.7.1.11, 3.1.3.11, 4.1.2.13, 1.2.1.12, 1.2.1.59, 2.7.2.3, 1.2.1.9, 1.2.7.6, 1.2.1.90, 5.4.2.11, 5.4.2.12, 4.2.1.11, 2.7.1.40
	ED	1.1.1.49, 3.1.1.31, 4.2.1.12, 4.1.3.42
	TCA	1.2.7.1, 2.3.3.1, 2.3.3.8, 4.2.1.3, 1.1.1.42, 1.1.1.41, 1.2.7.3, 1.2.7.11, 6.2.1.5, 6.2.1.4, 2.8.3.18, 1.3.5.1, 4.2.1.2, 1.1.5.4, 1.1.1.37
	PPP	5.3.1.9, 1.1.1.49, 3.1.1.31, 1.1.1.44, 1.1.1.343, 2.2.1.1, 5.3.1.6
	MP	2.7.2.1, 2.3.1.8, 6.2.1.1, 2.3.1.101, 3.5.4.27, 1.5.98.2, 2.1.1.246, 2.1.1.247, 2.1.1.248, 2.1.1.250, 2.1.1.86
Anabolism	CC	4.1.1.39, 2.7.2.3, 1.2.1.12, 1.2.1.59, 4.1.2.13, 3.1.3.11, 2.2.1.1, 3.1.3.37, 5.3.1.6
	WL	1.2.7.4, 2.3.1.169, 6.3.4.3, 3.5.4.9, 1.5.1.5, 2.1.1.258
	HY	6.4.1.2, 6.4.1.3, 1.1.1.298, 1.2.1.75, 6.2.1.36, 4.2.1.116, 1.3.1.84, 5.4.1.3, 4.2.1.148, 4.1.3.24, 4.2.1.153, 4.1.3.25, 5.1.99.1, 5.4.99.2, 1.2.1.76, 6.2.1.40, 4.2.1.120, 4.2.1.17, 1.1.1.35, 2.3.1.9, 2.8.3.22, 1.3.5.1, 4.2.1.2, 1.1.1.37, 4.1.1.31, 2.7.9.2
	MO	1.14.13.25, 1.14.18.3, 1.1.2.7, 1.1.3.13

The description and classification of ECs follow [37] and include only those ECs explicitly referenced in this study. ECs, Enzyme Commission numbers. EMP, Embden-Meyerhof-Parnas pathway; ED, Entner-Doudoroff pathway; TCA, Tricarboxylic acid cycle; PPP, pentose phosphate pathway; MP, Methanogenesis pathway; CC, Calvin cycle; WL, Wood-Ljungdahl pathway; HY, 3-Hydroxypropionate bi-cycle; MO, Methane oxidation pathway.

REFERENCES

1. Li D, Liu C-M, Luo R et al. MEGAHIT: an ultra-fast single-node solution for large and complex metagenomics assembly via succinct *de Bruijn* graph. *Bioinformatics* 2015;**31**:1674–6. https://doi.org/10.1093/bioinformatics/btv033
2. Hyatt D, Chen G-L, LoCascio PF et al. Prodigal: prokaryotic gene recognition and translation initiation site identification. *BMC Bioinformatics* 2010;**11**:119. https://doi.org/10.1186/1471-2105-11-119
3. Steinegger M, Söding J. MMseqs2 enables sensitive protein sequence searching for the analysis of massive data sets. *Nat Biotechnol* 2017;**35**:1026–8. https://doi.org/10.1038/nbt.3988
4. Buchfink B, Xie C, Huson DH. Fast and sensitive protein alignment using DIAMOND. *Nat Methods* 2015;**12**:59–60. https://doi.org/10.1038/nmeth.3176
5. Zheng B, Zhu Y, Sardans J et al. QMEC: a tool for high-throughput quantitative assessment of microbial functional potential in C, N, P, and S biogeochemical cycling. *Sci China Life Sci* 2018;**61**:1451–62. https://doi.org/10.1007/s11427-018-9364-7
6. Uritskiy GV, DiRuggiero J, Taylor J. MetaWRAP—a flexible pipeline for genome-resolved metagenomic data analysis. *Microbiome* 2018;**6**:158. https://doi.org/10.1186/s40168-018-0541-1
7. Parks DH, Imelfort M, Skennerton CT et al. CheckM: assessing the quality of microbial genomes recovered from isolates, single cells, and metagenomes. *Genome Res* 2015;**25**:1043–55. https://doi.org/10.1101/gr.186072.114

8. Langmead B, Salzberg SL. Fast gapped-read alignment with Bowtie 2. *Nat Methods* 2012;**9**:357–9. https://doi.org/10.1038/nmeth.1923

9. Tian J, Dungait JAJ, Hou R et al. Microbially mediated mechanisms underlie soil carbon accrual by conservation agriculture under decade-long warming. *Nat Commun* 2024;**15**:377–92. https://doi.org/10.1038/s41467-023-44647-4

10. Wen Y, Su LM, Qin WC et al. Linear and non-linear relationships between soil sorption and hydrophobicity: model, validation and influencing factors. *Chemosphere* 2012;**86**:634–40. https://doi.org/10.1016/j.chemosphere.2011.11.001

11. Xia Y, Chen X, Zheng X et al. Preferential uptake of hydrophilic and hydrophobic compounds by bacteria and fungi in upland and paddy soils. *Soil Biol Biochem* 2020;**148**:107879. https://doi.org/10.1016/j.soilbio.2020.107879

12. Barnett SE, Youngblut ND, Koechli CN et al. Multisubstrate DNA stable isotope probing reveals guild structure of bacteria that mediate soil carbon cycling. *Proc Natl Acad Sci USA* 2021;**118**:e2115292118. https://doi.org/10.1073/pnas.2115292118

13. LaRowe DE, Van Cappellen P. Degradation of natural organic matter: a thermodynamic analysis. *Geochim Cosmochim Acta* 2011;**75**:2030–42. https://doi.org/10.1016/j.gca.2011.01.020

14. Boye K, Noël V, Tfaily MM et al. Thermodynamically controlled preservation of organic carbon in floodplains. *Nat Geosci* 2017;**10**:415–9. https://doi.org/10.1038/ngeo2940

15. Barnett SE, Egan R, Foster B et al. Genomic features predict bacterial life history strategies in soil, as identified by metagenomic stable isotope probing. *mBio* 2023;**14**:e03584-22. https://doi.org/10.1128/mbio.03584-22

16. Kierans SJ, Taylor CT. Glycolysis: a multifaceted metabolic pathway and signaling hub. *J Biol Chem* 2024;**300**:107906. https://doi.org/10.1016/j.jbc.2024.107906

17. Chen H-P, Chow M, Liu C-C et al. Vanillin catabolism in rhodococcus jostii RHA1. *Appl Environ Microbiol* 2012;**78**:586–8. https://doi.org/10.1128/AEM.06876-11

18. Wu Z, Liang X, Li M et al. Advances in the optimization of central carbon metabolism in metabolic engineering. *Microb Cell Fact* 2023;**22**:76–86. https://doi.org/10.1186/s12934-023-02090-6

19. Kamimura N, Takahashi K, Mori K et al. Bacterial catabolism of lignin-derived aromatics: new findings in a recent decade: update on bacterial lignin catabolism. *Environ Microbiol Rep* 2017;**9**:679–705. https://doi.org/10.1111/1758-2229.12597

20. Yang S, Wu H, Wang Z et al. Linkages between the temperature sensitivity of soil respiration and microbial life strategy are dependent on sampling season. *Soil Biol Biochem* 2022;**172**:108758. https://doi.org/10.1016/j.soilbio.2022.108758

21. Razanamalala K, Razafimbelo T, Maron P-A et al. Soil microbial diversity drives the priming effect along climate gradients: a case study in Madagascar. *ISME J* 2018;**12**:451–62. https://doi.org/10.1038/ismej.2017.178

22. Morrissey EM, Mau RL, Schwartz E et al. Phylogenetic organization of bacterial

activity. *ISME J* 2016;**10**:2336–40. https://doi.org/10.1038/ismej.2016.28

23. Li H, Yang S, Semenov MV et al. Temperature sensitivity of SOM decomposition is linked with a K-selected microbial community. *Global Change Biol* 2021;**27**:2763–79. https://doi.org/10.1111/gcb.15593
24. Pepe-Ranney C, Campbell AN, Koechli CN et al. Unearthing the ecology of soil microorganisms using a high resolution DNA-SIP approach to explore cellulose and xylose metabolism in soil. *Front Microbiol* 2016;**7**:703. https://doi.org/10.3389/fmicb.2016.00703
25. Francioli D, Schulz E, Lentendu G et al. Mineral vs. Organic amendments: microbial community structure, activity and abundance of agriculturally relevant microbes are driven by long-term fertilization strategies. *Front Microbiol* 2016;**7**:1446. https://doi.org/10.3389/fmicb.2016.01446
26. Cederlund H, Wessén E, Enwall K et al. Soil carbon quality and nitrogen fertilization structure bacterial communities with predictable responses of major bacterial phyla. *Appl Soil Ecol* 2014;**84**:62–8. https://doi.org/10.1016/j.apsoil.2014.06.003
27. Yao F, Yang S, Wang Z et al. Microbial taxa distribution is associated with ecological trophic cascades along an elevation gradient. *Front Microbiol* 2017;**8**:2071. https://doi.org/10.3389/fmicb.2017.02071
28. Xu Q, Zhang H, Vandenkoornhuyse P et al. Carbon starvation raises capacities in bacterial antibiotic resistance and viral auxiliary carbon metabolism in soils. *Proc Natl Acad Sci USA* 2024;**121**:e2318160121.

https://doi.org/10.1073/pnas.2318160121

29. Donhauser J, Qi W, Bergk-Pinto B et al. High temperatures enhance the microbial genetic potential to recycle C and N from necromass in high-mountain soils. *Global Change Biol* 2021;**27**:1365–86. https://doi.org/10.1111/gcb.15492

30. Auer E, Lazuka A, Huguenin-Bizot B et al. Horizontal metaproteomics and CAZymes analysis of lignocellulolytic microbial consortia selectively enriched from cow rumen and termite gut. *ISME Commun* 2023;**3**:134–45. https://doi.org/10.1038/s43705-023-00339-0

31. Dai Z, Zang H, Chen J et al. Metagenomic insights into soil microbial communities involved in carbon cycling along an elevation climosequences. *Environ Microbiol* 2021;**23**:4631–45. https://doi.org/10.1111/1462-2920.15655

32. Cardenas E, Kranabetter JM, Hope G et al. Forest harvesting reduces the soil metagenomic potential for biomass decomposition. *ISME J* 2015;**9**:2465–76. https://doi.org/10.1038/ismej.2015.57

33. Huang Q, Wang B, Shen J et al. Shifts in C-degradation genes and microbial metabolic activity with vegetation types affected the surface soil organic carbon pool. *Soil Biol Biochem* 2024;**192**:109371. https://doi.org/10.1016/j.soilbio.2024.109371

34. López-Mondéjar R, Tláskal V, Větrovský T et al. Metagenomics and stable isotope probing reveal the complementary contribution of fungal and bacterial communities in the recycling of dead biomass in forest soil. *Soil Biol Biochem* 2020;**148**:107875. https://doi.org/10.1016/j.soilbio.2020.107875

35. Liang J, Chang J, Zhang R et al. Metagenomic analysis reveals the efficient digestion mechanism of corn stover in angus bull rumen: microbial community succession, CAZyme composition and functional gene expression. *Chemosphere* 2023;**336**:139242. https://doi.org/10.1016/j.chemosphere.2023.139242

36. Bredon M, Dittmer J, Noël C et al. Lignocellulose degradation at the holobiont level: teamwork in a keystone soil invertebrate. *Microbiome* 2018;**6**:162. https://doi.org/10.1186/s40168-018-0536-y

37. Wang N, Li K, Yuan F et al. Faster cycling but lower efficiency: a microbial metabolic perspective on carbon loss after wetland conversion to cropland. *Soil Biol Biochem* 2024;**189**:109260. https://doi.org/10.1016/j.soilbio.2023.109260
